# Fine-Tuning Amyloid Precursor Protein Expression through Nonsense-Mediated mRNA Decay

**DOI:** 10.1523/ENEURO.0034-24.2024

**Published:** 2024-06-03

**Authors:** Maryam Rahmati, Jasmine Chebli, Rakesh Kumar Banote, Sandra Roselli, Lotta Agholme, Henrik Zetterberg, Alexandra Abramsson

**Affiliations:** ^1^Department of Psychiatry and Neurochemistry, Institute of Neuroscience and Physiology, The Sahlgrenska Academy, University of Gothenburg, Gothenburg 413 45, Sweden; ^2^Department of Neurodegenerative Disease, UCL Institute of Neurology, Queen Square, London WC1N #BG, United Kingdom; ^3^Clinical Neurochemistry Laboratory, Sahlgrenska University Hospital, Mölndal 431 41, Sweden; ^4^United Kingdom Dementia Research Institute, London W1T 7NF, United Kingdom; ^5^Hong Kong Center for Neurodegenerative Diseases, 17 Science Park W Ave, Hong Kong, China; ^6^Wisconsin Alzheimer’s Disease Research Center, University of Wisconsin School of Medicine and Public Health, University of Wisconsin-Madison, Madison, Wisconsin 53792

**Keywords:** amyloid precursor protein, mutant, NMD, transcriptional adaptation, Upf1, zebrafish

## Abstract

Studies on genetic robustness recently revealed transcriptional adaptation (TA) as a mechanism by which an organism can compensate for genetic mutations through activation of homologous genes. Here, we discovered that genetic mutations, introducing a premature termination codon (PTC) in the amyloid precursor protein-b (*appb*) gene, activated TA of two other *app* family members, *appa* and amyloid precursor-like protein-2 (*aplp2*), in zebrafish. The observed transcriptional response of *appa* and *aplp2* required degradation of mutant mRNA and did not depend on Appb protein level. Furthermore, TA between amyloid precursor protein (APP) family members was observed in human neuronal progenitor cells; however, compensation was only present during early neuronal differentiation and could not be detected in a more differentiated neuronal stage or adult zebrafish brain. Using knockdown and chemical inhibition, we showed that nonsense-mediated mRNA decay (NMD) is involved in degradation of mutant mRNA and that Upf1 and Upf2, key proteins in the NMD pathway, regulate the endogenous transcript levels of *appa*, *appb*, *aplp1*, and *aplp2*. In conclusion, our results suggest that the expression level of App family members is regulated by the NMD pathway and that mutations destabilizing *app*/*APP* mRNA can induce genetic compensation by other family members through TA in both zebrafish and human neuronal progenitors.

## Significance Statement

Genetic variations increasing amyloid precursor protein (APP) levels are associated with Alzheimer's disease pathophysiology. It is therefore of key interest to understand the mechanisms regulating APP expression. Here, we identify transcriptional adaptation as a mechanism by which members of the APP family can modulate the expression level of genes in the same family to compensate for the loss of another. Upon the introduction of a PTC, compensation is driven through nonsense-mediated mRNA decay (NMD). Interestingly, our data also show that the NMD surveillance machinery is an important aspect of fine-tuning mRNA levels of APP family members even under physiological conditions. Our findings provide insights into compensation between APP members and reveal new targets by which APP can be regulated.

## Introduction

Understanding gene function has fascinated scientists since DNA and the transfer of genes between generations were described. Inbreeding, or forward genetics, by which a phenotype of interest was searched among individuals with random genome-wide mutations or by generating targeted genetic mutations, has been used to elucidate gene function. While the role of some genes has been easy to assess, others have been challenging due to redundant gene function and compensatory mechanisms. One such example is the amyloid beta (A4) precursor protein (App) and the amyloid precursor-like protein (Aplp)-1 and Aplp2 in mice, whose single mutant gene knockouts show only minor phenotypes, while combined knock-out mutations are lethal (von Koch et al., 1997; Heber et al., 2000). While redundancy allows one protein to functionally replace another protein if lost, genetic compensation ensures adjusted expression levels. This type of genetic robustness is an essential system enhancing survival of organisms despite harmful mutations (Kitano, 2004; Felix and Barkoulas, 2015). Recent studies support the existence of a new mechanism governing genetic robustness, described as transcriptional adaptation (TA) or genetic compensation response (GCR; Kimmel et al., 1995; Zhu et al., 2017; El-Brolosy et al., 2019; Ma et al., 2019). These findings came from studies dissecting the phenotypic discrepancies observed between morpholino-antisense oligomer (MO) knockdowns and genetic mutants, originally thought of as MO-induced toxicity or off-target effects (Kok et al., 2015; Rossi et al., 2015). Interestingly, while both TA and GCR were reported as activated by premature termination codon (PTC) mutations, mRNA degradation is important to trigger TA (El-Brolosy et al., 2019). Most PTC-containing transcripts are degraded through nonsense-mediated mRNA decay (NMD), providing an efficient mechanism by which aberrant and toxic protein synthesis in cells are removed. Interestingly, NMD not only serves to scavenge nonfunctional mRNA but also fine-tunes physiologically normal mRNAs (Mendell et al., 2004; Wittmann et al., 2006; McIlwain et al., 2010; Hurt et al., 2013; Kurosaki et al., 2019; Yi et al., 2021). Thus, dysfunction in the NMD proteins is related to pathological conditions including neurodegeneration, neurodevelopmental disorders, and cancer (Giorgi et al., 2007; Bruno et al., 2011; Colak et al., 2013; Lou et al., 2014).

The NMD core protein complex consists of the up-frameshift proteins Upf1, Upf2, and Upf3 (Wang et al., 2001; Lejeune and Maquat, 2005; Amrani et al., 2006). In eukaryotes, there are two Upf3 paralogs, Upf3a and Upf3b, with both overlapping and distinct functions (Bushman et al., 2015; Shum et al., 2016). The Upfs are conserved throughout eukaryotes and serve to detect premature translation events upstream of the normal termination site. Although not fully understood, the classic complex consists of Upf1, the main degradation factor that binds randomly to mRNA and becomes activated by suppressor of morphogenesis in genitalia-1 (SMG1); Yamashita, 2013). This process is promoted by the interaction with Upf2 and Upf3. However, data now support that the outcome of NMD depends on the assembling subunits, which in part relies on whether the complexes interact or not with the exon junction complex (EJC; Bushman et al., 2015; Yi et al., 2021). The EJC, deposited at exon–exon boundaries of spliced mRNAs, are removed by the ribosomes during the first round of translation. In the event of a PTC, the downstream EJC remains and binds the Upf3b–Upf2 complex by recruiting SMGs to promote Upf1 interaction and RNA decay (Melero et al., 2014; Bufton et al., 2022). Alternatively, Upf1 can bind directly to long 3′-UTRs, without interacting with Upf3b-EJC, which increases the likelihood of NMD (Hurt et al., 2013; Gao and Wilkinson, 2017).

Considering these new findings, we set out to address if mutations in *APP* induce TA of other *APP* family members as a mechanism to activate compensation. We first investigated if TA caused the phenotypic difference observed in the partial knockdown of *appb* using MO (Joshi et al., 2009; Abramsson et al., 2013; Banote et al., 2016) compared with the CRISPR/Cas9-generated genetic *appb* mutant (shown as *appb^–/–^*; Banote et al., 2020). Like mice and humans, zebrafish harbor genes coding for *aplp1* and *aplp2*, while the partial third genome duplication gave rise to two *APP* orthologs, *appa* and *appb* (Nicolas and Hassan, 2014). To confirm that degradation of faulty mRNA is important to trigger TA, we generated the RNA-less *appb* mutant (shown as *appb^P–/–^*) lacking *appb*. We then investigated if TA within the APP gene family is conserved in human neuronal cells. Finally, to reveal the mechanism behind the TA between App family members, we inhibited or knocked down NMD core proteins and measured expression level of App family members.

## Materials and Methods

### Animal care and ethics statement

Zebrafish (*Danio rerio*) were maintained in Aquatic Housing Systems (Aquaneering) on a 14/10 h light/dark cycle at 28.5°C, at the facility of the Institute of Neuroscience and Physiology, University of Gothenburg. NaHCO_3_ and coral sand were used to keep the system water at a pH between 7.2 and 7.4 and Instant Ocean salt to sustain the conductivity at 900 μS. Larva were raised in embryo medium (EM; in mM: 1.0 MgSO_4_, 0.15 KH_2_PO_4_, 0.042 Na_2_HPO_4_, 1 CaCl_2_, 0.5 KCl, 15 NaCl, 0.7 NaHCO_3_) at 28.5°C in a dark incubator. Fish were fed Gemma granular fish food (Skretting) twice a day and live brine shrimp (*Artemia*) or marine L-type rotifers (*Brachionus plicatilis*, ZM Systems). Staging of fish embryos was carried out according to hours postfertilization (hpf) or days postfertilization (dpf; Kimmel et al., 1995). For all the experiments, 0.02% tricaine methanesulfonate (MS-222, Sigma Aldrich) was used to anesthetize larva before experiment. All animal procedures were performed in accordance with the University of Gothenburg animal care committee's regulations. All studies involving zebrafish were performed in accordance with local guidelines and approved by regional authorities. The experiments used AB wild-type fish and the previously published *appb^26_2^* mutants abbreviated as *appb^−/−^* (Banote et al., 2020). The *appb^P−/−^* (*appb^P17^*) fish line was generated in this study. Fish of either gender was used in the experiments.

### Mutagenesis using the CRISPR/Cas9 system

Generation of RNA-less mutants was performed using the CRISPR/Cas9 system, as previously described (Varshney et al., 2016). Guide RNAs (gRNAs) were designed to delete approximately 500–1,000 base pairs (bp) of the *appb* gene, depending on the position of gRNAs, including the ATG site. A 1,129 base-pair sequence, located 127 bp downstream and 1,002 bp upstream of the ATG, was used for blasting with CRISPOR online tool (www.crispor.tefor.net) to design gRNAs, including the 5′-UTR region and exon 1 of *appb*. Twelve different target-specific DNA oligos were selected based on their GC content, specificity score, and the number of possible off-targets. A modified version of target DNA was ordered with additional GG motif in the beginning of target sequence to facilitate RNA polymerase binding and more efficient in vitro transcription (Extended Data Table 4-1). Guide RNAs were named based on the location of the PAM sequence with respect to the contig used during the research where a low number is upstream of the ATG site and the highest numbers are downstream of the ATG site. gRNAs were synthesized by assembling each target-specific DNA oligomer with a “generic” DNA oligomer (5′-AAAAGCACCGACTCGGTGCCACTTTTTCAAGTTGATAACGGACTAGCCTTATTTTAACTTGCTATTTCTAGCTCTAAAAC-3′) coding for the guide RNA. The two oligos were annealed and extended with *Pfu Ultra* High-Fidelity DNA Polymerase (Agilent) to produce a double-stranded DNA fragment. The resulting product serves as a template for in vitro transcription using T7 Quick High Yield RNA Synthesis Kit (New England Biolabs). Embryos were coinjected with 50 pg gRNA and 300 pg Cas9 protein (Integrated DNA Technologies), into the yolk at the one-cell stage. Injected embryos were screened for gRNA activity using T7 Endonuclease I assay (Mashal et al., 1995) using specific reverse and forward primers to amplify the target site (Extended Data Table 4-2). The confirmed two active gRNAs were coinjected into wild-type zebrafish, and the embryos were raised to adulthood. Each founder (F0) was outcrossed with wild-type zebrafish of AB background. Ten embryos were selected; a 1,212 bp region surrounding the target site was amplified by PCR using a forward primer (5′-TGTCATGCGTTTTCCCTTCAC-3′) and a reverse primer (5′-CTTATCCAGCCCTTCCAGTCG-3′). Gel electrophoresis and Sanger sequencing were performed to identify the founders carrying a deletion between the two gRNAs used. The remaining embryos of the confirmed founders carrying promoter deletions were raised to adulthood (F1). Fin clipping was performed on F1 generation to extract DNA and identify heterozygotic individuals. Identified heterozygotes carrying the mutant allele were outcrossed with wild-type AB zebrafish for two generations and then inbred to generate homozygotes. Sanger sequencing of homozygous mutants and wild-type zebrafish was performed on genomic DNA from fin clips using DNeasy Blood & Tissue Kits (Qiagen). In short, genomic DNA was amplified using the mentioned forward primer and reverse primer. The amplified product was cleaned with FastAP and *exonuclease I* (Thermo Fisher Scientific) according to the manufacturer's instructions. Sanger sequencing with BigDye Terminator v1.1 Cycle Sequencing Kit (Applied Biosystems) on an ABI3130xl sequencer (SeqGen) revealed a large deletion of 979 bp between the sites of used gRNAs with 7 bp added, resulting in a total deletion of 972 bp between position −918 and +61 if considering A of ATG as +1. All the experiments in this study have been performed on the F2 and F3 generation.

### Capped and uncapped RNA transcription

Since we were not able to clone the *appb* mutant cDNA from *appb^−/−^* larvae due to extensive RNA degradation, we instead introduced the mutation in a previously cloned *appb* wild-type cDNA using *PfuUltra* High-Fidelity DNA Polymerase (Agilent) and the QuikChange Primer Design software to design primers for mutagenesis. The following primers were used to introduce the *appb^−/−^* mutation; Fwd 5′-TCGGTGGGCTCAGGAGCCTCAGGTGGCCATG-3′ and Rev 5′-CTGAGGCTCCTGAGCCCACCGAGTCATCCG-3′. The wild-type *appb* cDNA in pcDNA3 (Abramsson et al., 2013) vector was amplified, original plasmid removed by *DpnI* (New England Biolabs) digestion for 3 h, and then transformed into One Shot Top10 Chemically Competent *E. coli* (Thermo Fisher Scientific). The mutation was verified by Sanger sequencing. The pSYC-97 plasmid (pCS4+-NLS-EGFP-P2A-mCherry-CAAX) was a gift from Seok-Yong Choi (Addgene plasmid #31565) and was used as the control plasmid (Kim et al., 2011). pcDNA3 and pSYC-97 plasmids were linearized using *NaeI* (Biolabs) and *NotI* (Roche) and then in vitro transcribed using mMESSAGE mMACHINET7 Ultra Transcription kit (Invitrogen) and the mMESSAGE mMACHINE SP6 promoter kit (Invitrogen), respectively. To transcribe uncapped RNAs, 20 µl reaction containing 1 µg linearized DNA, 1× transcription buffer, 1× DIG-dNTP, 20 U T7 polymerase, and 20 U RNase inhibitor (all from Roche) was made and incubated for 2 h at 37°C. After incubation, RNA was treated with 4U DNase TURBO (Ambion) and incubated for 15 min at 37°C. The reaction was stopped with 0.5 M EDTA. All RNAs were purified using RNA Clean & Concentrator-5 from Zymolab and diluted to 25–100 ng/μl for injection.

### Pharmacological treatments

To inhibit NMD, we treated 24 hpf *appb^−/−^* mutant larvae with 10 µM NMDi14 (MedChemExpress) or with 0.1% DMSO (Thermo Fisher Scientific), and 24 h later they were collected and snap frozen for RNA extraction. To block translation, we treated 22–24 hpf *appb^−/−^* mutants with EM with or without 15 µg/ml cycloheximide (CHX; Sigma Aldrich). After 5 h, embryos were collected and snap frozen for RNA extraction. For each sample, 10 larvae per well were treated in a 24-well plate. The experiments were done in three biologically independent replicates and each round included five technical replicates.

### Microinjection of morpholinos and mRNA

The morpholino antisense oligomers (Gene Tools) targeting the *appb* translation start (5′-TGTGTTCCCAAGCGCAGCACGTCCT-3′) or splicing donor of exon 2 (5′-CTCTTTTCTCTCTCATTACCTCTTG-3′) were injected at the one-cell stage using 1 ng for Mauthner cell analysis and 2.5 ng for qPCR and Western blot analysis (Banote et al., 2016). For knocking down the NMD pathway, *upf1*MO (5′-CGCCTCCACACTCATCTTTATATTC-3′), *upf2*MO (5′-ATGCACTACAGCACTCACATGAAAT-3′), and *upf3a*MO (5′-TCTGCTCCTTTTCAGACCTCATATC-3′) were designed as described in previous studies (Wittmann et al., 2006; Ma et al., 2019). *upf3b*MO (5′-ATCGAGGTTTGGCTTTACCAGACAT-3′) was designed to target the splicing site of exon 3 and intron 3, and its effectiveness was tested by PCR analysis (Extended Data Fig. 6-1). Five nanograms of *upf1*MO and *upf2*MO and 1 ng each of *upf3a*MO and *upf3b*MO were injected into fertilized embryos at the one-cell stage. The following primers were used to show the efficiency of *upf3b* knockdown: Fwd 5′-CGCTTCGATGGCTATGTCTTCA and Rev 5′-ACTTACGCCGTTCTTCCTCTCG. Amplification was performed using Taq DNA Polymerase (Invitrogen) with the following conditions: 95°C for 3 min for initiation, continued with a 40 times repeating cycle of 20 s at 95°C, 40 s at 67°C, 25 s at 68°C, and 5 min at 68°C for the final extension step (Extended Data Fig. 6-1). For injections, borosilicate injection needles were pulled using P-97 Flaming/Brown micropipette puller (Sutter Instrument). All injections were performed with a FemtoJet microinjector (Eppendorf). All morpholinos were purchased from Gene Tools.

### Immunostaining and Mauthner cell imaging

Staining of the Mauthner cells was performed as described previously (Banote et al., 2016). Embryos were incubated in 0.02% 1-phenyl-2-thiourea (PTU, Sigma Aldrich) at 22 hpf to prevent pigmentation. For immunostaining of neurofilament, embryos were anesthetized and fixed in 2% trichloroacetic acid (Sigma Aldrich) at 48 hpf for 3 h at room temperature, then washed in phosphate-buffered saline (PBS), and blocked in 0.5% Triton X-100, 10% normal goat serum, and 0.1% bovine serum albumin (BSA) in PBS for 1 h at room temperature. Antibody labeling was performed using monoclonal mouse anti-neurofilament RMO44 antibody (Sigma Aldrich) followed by goat anti-mouse Alexa Fluor 488 (Invitrogen) as secondary antibody at 1:1,000 and 1:500 dilutions, respectively, and incubated overnight (ON) at 4°C. Brains were dissected, mounted in 1% low temperature gelling agarose (Sigma Aldrich, A4018) in glass bottom dishes (Cellvis), and imaged using Zeiss LSM710 confocal microscope (Carl-Zeiss). Images were analyzed and produced using ImageJ software (National Institutes of Health).

### Western blot

Protein was extracted at 3 dpf from wild-type (WT), *appb*MO translation blocker, *appbMO* splicing blocker, and *appb^P−/−^* mutant larvae (60 larvae per *n*; *n* = 3) to confirm the loss of protein in mutants. Larvae were killed, deyolked in cold EM and snap frozen in liquid nitrogen and stored in −80°C before use. Samples were thawed on ice and homogenized in an ice-cold lysis buffer (10 mM Tris–HCl, pH 8.0, 2% sodium deoxycholate, 2% SDS, 1 mM EDTA, 0.5 M NaCl, 15% glycerol) mixed with protease inhibitors cocktail (Roche) using a 23 G syringe. Homogenized samples were incubated for 20 min on ice and sonicated at maximum intensity for 10 min. After sonication, samples were incubated for 30 min on ice and centrifuged at 10,000 × *g* at 4°C. The supernatant was transferred into a new 1.5 ml Eppendorf tube and protein concentration measured with a BCA Protein Assay Kit (Thermo Fisher Scientific). Proteins were separated on a NuPAGE NOVEX Bis–TRIS pre-cast gel (Thermo Fisher Scientific) and transferred onto a transfer blot-turbo membrane (Bio-Rad). The membrane was incubated in a 5% milk as a blocking solution for 2 h at RT and then immunoblotted with the polyclonal *appb*-specific antibody (EER15; 1:10,000), an inhouse-generated polyclonal rabbit anti-Appb antibody against N-terminal half of Aβ peptides (Banote et al., 2020), overnight at 4°C. To detect Appb, we then washed the membrane in TBS-Tween 3 × 10 min at RT and incubated with the secondary antibody IRDye 800CW Goat anti-rabbit (1:10,000) IgG (LI-Cor Biotechnology). The membrane was stripped with stripping buffer for 10 min (Thermo Fisher Scientific), washed in TBS-Tween 3 × 10 min, and then blocked for 1 h in 5% milk as the blocking solution. The membrane was then incubated with a loading concentration control mouse anti-GAPDH-HRP conjugated (1:10,000; Novus Biologicals) for 1 h followed by 3 × 10 min washes in TBS-Tween. The signal was developed using the SuperSignal West Dura Extended Duration Substrate Kit (Thermo Fisher Scientific) and imaged using ChemiDoc Imaging Lab program (Bio-Rad). Quantifications were performed by ImageJ Fiji software (NIH). Band intensities were normalized to loading control and shown as relative controls.

### RNA extraction from whole zebrafish larvae and quantitative PCR

To show the mRNA level of *app* family members, RNA was extracted from whole larvae at 24 hpf. Embryos of AB or *appb^+/+^* background were used as controls. Each experiment was performed at least three times with four to five technical replicates (considered as “*N*”) of 10 larvae each. Total RNA was extracted from 10 larvae, using TRI Reagent (Sigma Aldrich). Then, RNA samples were treated with RQ1 1× RNase-free DNase reaction buffer and RQ1 RNase-free DNase (Promega kit). cDNA was synthesized using High-Capacity RNA-to-cDNA Kit (Applied Biosystems) and converted in a single-cycle reaction on a 2,720 Thermal Cycler (Applied Biosystems). Quantitative PCR (qPCR) was performed with inventoried TaqMan Gene Expression Assays with FAM reporter dye (Thermo Fisher Scientific) in TaqMan Fast Advanced Master Mix (Applied Biosystems). The assay was carried out on Micro-Amp 96-well optical microtiter plates on a QuantStudio 3 Real-Time PCR System Software (Applied Biosystems). qPCR results were analyzed with the QuantStudio Design and Analysis software v1.5.2 (Applied Biosystems). Briefly, C_T_ from each sample was normalized with average C_T_:s of *eef1a1l1* and *actb1*, and then the relative quantity was determined using the ΔΔC_T_ method (Livak and Schmittgen, 2001) with a sample of wild-type embryos (6–72 hpf and adult brain) as the calibrator. TaqMan Gene Expression Assays (Applied Biosystems) were used for the following genes: Amyloid Beta (A4) Precursor Protein A (*appa*, Dr 03144364_m1 and Dr 03144365_m1), Amyloid Beta (A4) Precursor Protein B (*appb*, Dr 03080308_m1 and Dr 03080304_m1), Amyloid Beta Precursor Like Protein 1 (*aplp1*, AJCSWD2), Amyloid Beta Precursor Like Protein 2 (*aplp2*, Dr 03437773_m1), Eukaryotic Translation Elongation Factor 1 Alpha 1, Like 1 (*eef1a1l1*, Dr 03432748_m1), and Actin Beta 1 (*actb1*, Dr 03432610_m1). To confirm the deletion of *appb* in *appb^P−/−^*, SYBR green primers were designed to amplify exon 16–17 in *appb* and *eef1a1* primer pairs used as housekeeping gene (Extended Data Table 4-3).

### RNA sequencing

RNA extracted from whole zebrafish larvae was performed as described above. The quantity and quality of isolated RNA was determined using a 4200 TapeStation Automated Electrophoresis System (Agilent Technologies). All samples had an RNA integrity number >9.3. Sequencing libraries were prepared using the Illumina Stranded mRNA prep kit (Illumina), following the manufacturer's instructions. Construction of libraries were prepared using the Illumina Stranded mRNA Prep Guide 1000000124518 v02 (Illumina), using 500 ng of total RNA input. The Novaseq 6000 platform was used, and 150 bp paired-end reads were generated by Clinical Genomics.

### Bioinformatic analysis

To control the quality of the sequencing data, a multiqc report, version 1.13, was generated. Refseq and Ensembl reference genomes for *D. rerio* were collected from the NCBI and Ensembl online databases. Indexing of reference genomes as well as alignment was performed using STAR version 2.7.10b (Dobin et al., 2013). To improve the alignment of the novel splice junction, STAR was run with the two-pass Mode argument (Veeneman et al., 2016). BAM files were indexed by SAMtools (Danecek et al., 2021) version 1.9 whereafter BAM and index files were used for visualization in IGV version 2.16.2.

Sashimi plots were generated using Integrative Genomics Viewer 2.16. Minimum Junction coverage was set between 9 and 30.

To discover any splicing variants, PSI-sigma version 2.1 (Lin and Krainer, 2019) was run as well as StringTie version 2.2.1 (https://ccb.jhu.edu/software/stringtie/).

Manual assembly of the region spanning *appa* was performed by searching for contigs matching the exon 11–12 of *appa* (NM_131564.2) in Refseq using Blastn with search parameters set to the whole-genome shotgun contigs (WGS), *D. rerio* as the organism and searching for highly similar sequences. Exon 11–12 mapped 100% to the contig JALCZS010004005.1. Our RNAseq data and previously cloned *appa* cDNA indicated the existence of an additional exon between 11 and 12. The new exon 12 (marked in blue in Extended Data Fig. 7-1) showed a 100% sequence alignment with contig LKPD02013461.1 that was included within JALCZS010004005.1. The contig JALCZS010004005.1 aligned to both FP067437.2 and FO704780.1. A fully annotated sequence of the region can be shared if requested.

Trinity (Grabherr et al., 2011) version 2.15.1 was run to create a de novo transcriptome using the reads from the three wild-type samples. Alignment of the WT control reads to the de novo transcriptome was performed by running STAR, leaving out –sjdbGTFfile –twopassMode arguments, as this was done in order to estimate the read content of the de novo assembly. The manually created sequence was aligned to the de novo assembly using BLAT version 35. Furthermore, BLAT was run to investigate alignment between an Ensembl reference transcriptome (https://ftp.ensembl.org/pub/release-111/fasta/danio_rerio/cdna/) and the de novo transcriptome generated by trinity.

### Cell culture and transfection

Cortical neural progenitor cells (NPCs) and neurons were differentiated from two lines of huma-induced pluripotent stem cells (hiPSCs), Ctrl1 (Sposito et al., 2015) and ChiPSC22 (Cellartis by Takara Bio Europe), using a modified version of the protocol from Shi et al. (2012). The detailed neural differentiation procedure is described before (Bergstrom et al., 2016). NPCs were cultured on human laminin L521 (0.5 µg/cm^2^, BioLamina) until around 35 d after induction, and then further differentiated into neurons for 35 more days on poly-L ornithine (0.01%, Sigma Aldrich) and human laminin L521 (0.5 µg/cm^2^). All cell cultures were kept in a humidified atmosphere at 5% CO^2^ and 37°C.

The day before transfection, NPCs (27–29 d after induction) and terminally differentiated neurons (70 d after induction) were passaged with StemPro Accutase (Thermo Fisher Scientific) and seeded at a density of 100k/cm^2^. The hAPP_695_:GFP-N1 pcDNA3.1 plasmid, kindly provided by Olav Andersen (Aarhus, Denmark; Andersen et al., 2005), was linearized and in vitro transcribed as described above. Cells were transfected with 500 ng uncapped h*APP* in a 24-well plate using Lipofectamine MessengerMAX (Thermo Fisher Scientific) transfection reagents according to the manufacturer's protocol. Twenty-four hours after transfection, cells in each well were collected in 350 μl RNeasy Lysis Buffer (RLT) buffer containing dithiothreitol (DTT), and RNA was extracted using RNeasy Mini kit (Qiagen) according to the manufacturer's protocol. cDNA was synthetized and qPCR was performed as described above. *RPLP27* and *HPRT1* were used as housekeeping genes. Untreated cells were used as calibrator. TaqMan Gene Expression Assays (Applied Biosystems) were used for the following genes: Amyloid Beta (A4) Precursor Protein (*APP*, Hs00169098_m1), Amyloid Beta Precursor Like Protein 1 (*APLP1*, Hs00193069_m1), Amyloid Beta Precursor Like Protein 2 (*APLP2*, Hs00155778_m1), Receptor Like Protein 27 (*RLP27*, Hs03044961_g1), and Hypoxanthine Phosphoribosyltransferase 1 (*HPRT1*, Hs02800695_m1).

### Illustrations

**Figure 1.**
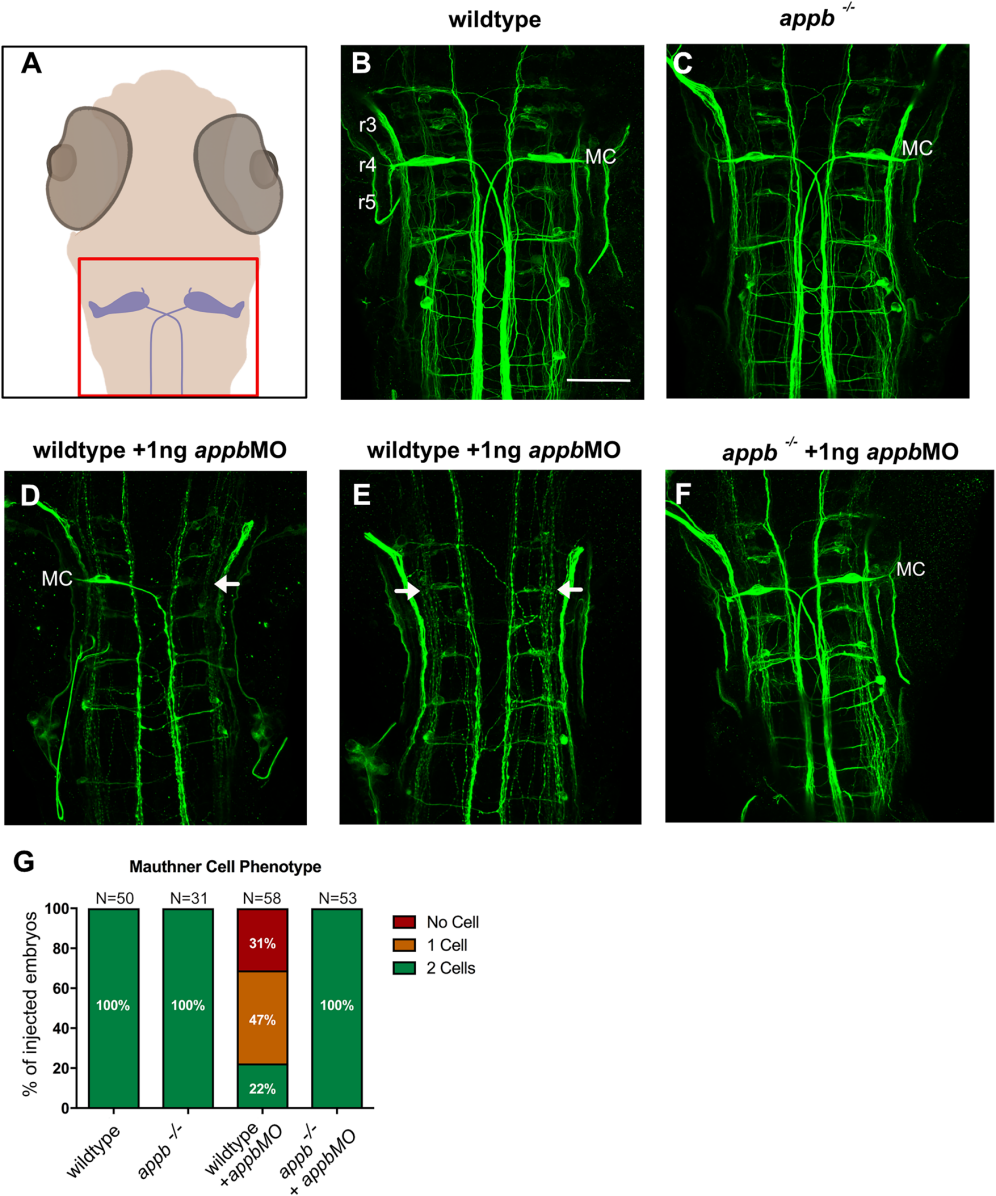
*appb*MO-mediated loss of Mauthner cells in wild type but not *appb^−/−^* zebrafish. ***A***, Schematic image of the hindbrain region shown in ***B–F***. ***B***, ***C***, Dorsal view of hindbrain of embryos (anterior to the top) stained with RMO44 antibody at 48 hpf, displaying the large Mauthner cells (MC) in wild type (***B***) and *appb^−/−^* (***C***). ***D***, ***E***, Wild-type larvae injected with 1 ng splice-blocking *appb*MO showed two (22%), one (47%, ***D***), or no MC (31%, ***E***). ***F***, two MC were observed in all *appb^−/−^* injected with 1 ng splice-blocking *appb*MO. ***G***, Quantification of the MC number at 48 hpf. ***G***, *n* = 3 biologically independent samples. “*N*” indicates number of brains. r3–r5, rhombomeres 3–5. Scale bar, 50 μm. MC, Mauthner cells. Arrows indicate missing Mauthner cells.

Figure 1*A* made by J.C. using Affinity Designer (Serif Europe, Apple). Figures 6*A* and 10 (illustration of conclusion) were created in Biorender.com.

### Statistical analysis

Statistical analysis was performed using GraphPad Prism 9 software (Prism). Continuous data were presented using mean and standard deviation of the mean (±SD). Total number of individual samples is shown as “*N*” and the number of biological independent replicates as “*n*.” Results were compared statistically using two-tailed Student's *t* tests unless stated otherwise. Statistical significance was set at *p < 0.05, **p < 0.01, ***p < 0.005, ****p < 0.0001.

## Results

### Discrepancy between knockdown and knock-out phenotypes is not due to morpholino off-target effects

Functional redundancy between *App* family members is generally accepted as the underlying mechanism behind the lack of major phenotypes in single-gene-knock-out mice (Shariati and De Strooper, 2013). However, if such redundancy also engages genetic compensation is not yet known. Similar to many other zebrafish mutants (Kok et al., 2015; Rossi et al., 2015; Morgens et al., 2016; Housden et al., 2017), the genetic *appb* mutants (Banote et al., 2020) show a milder phenotype than the *appb* knockdowns (Joshi et al., 2009; Abramsson et al., 2013; Banote et al., 2016). To evaluate the phenotypic discrepancy between knockdown and knock-out, we began by addressing the specificity of the *appb* antisense morpholino (*appbMO*). As previously reported, knockdown of *appb* results in defect convergence and extension, curved trunk, and partial or complete loss of Mauthner cells (MC) in zebrafish embryos (Joshi et al., 2009; Banote et al., 2016). We used the previously reported *appb^−/−^*, harboring a PTC in exon 2 (Banote et al., 2020), to study the genetic loss of *appb*. Immunostaining of *appb^−/−^* using an antibody against neurofilament (RMO44) showed the presence of two bilaterally positioned MCs like wild-type embryos (Fig. 1*A–C*). We reasoned that if the loss of MCs in *appb* knockdown larvae was an off-target effect, then this phenotype should be preserved in the *appbMO*-injected *appb^−/−^*. To this end, we evaluated MC formation in wild-type and *appb^−/−^* embryos injected with an *appb* antisense morpholino (*appbMO*; Fig. 1*D–F*). Interestingly, while MCs were absent unilaterally (∼31%) or bilaterally (∼47%) in the *appbMO*-injected wild-type embryos, two MCs were present in all *appbMO*-injected *appb^−/−^* at 48 h postfertilization (hpf; Fig. 1*G*). Thus, the lack of MC defects in *appb^−/−^* indicates an *appb*-specific effect of the *appbMO* and that the *appb* mutation masks the morpholino phenotype.

### Upregulated expression of *app* family members in genetic *appb^−/−^* but not in morpholino-mediated *appb* knockdown

**Figure 2.**
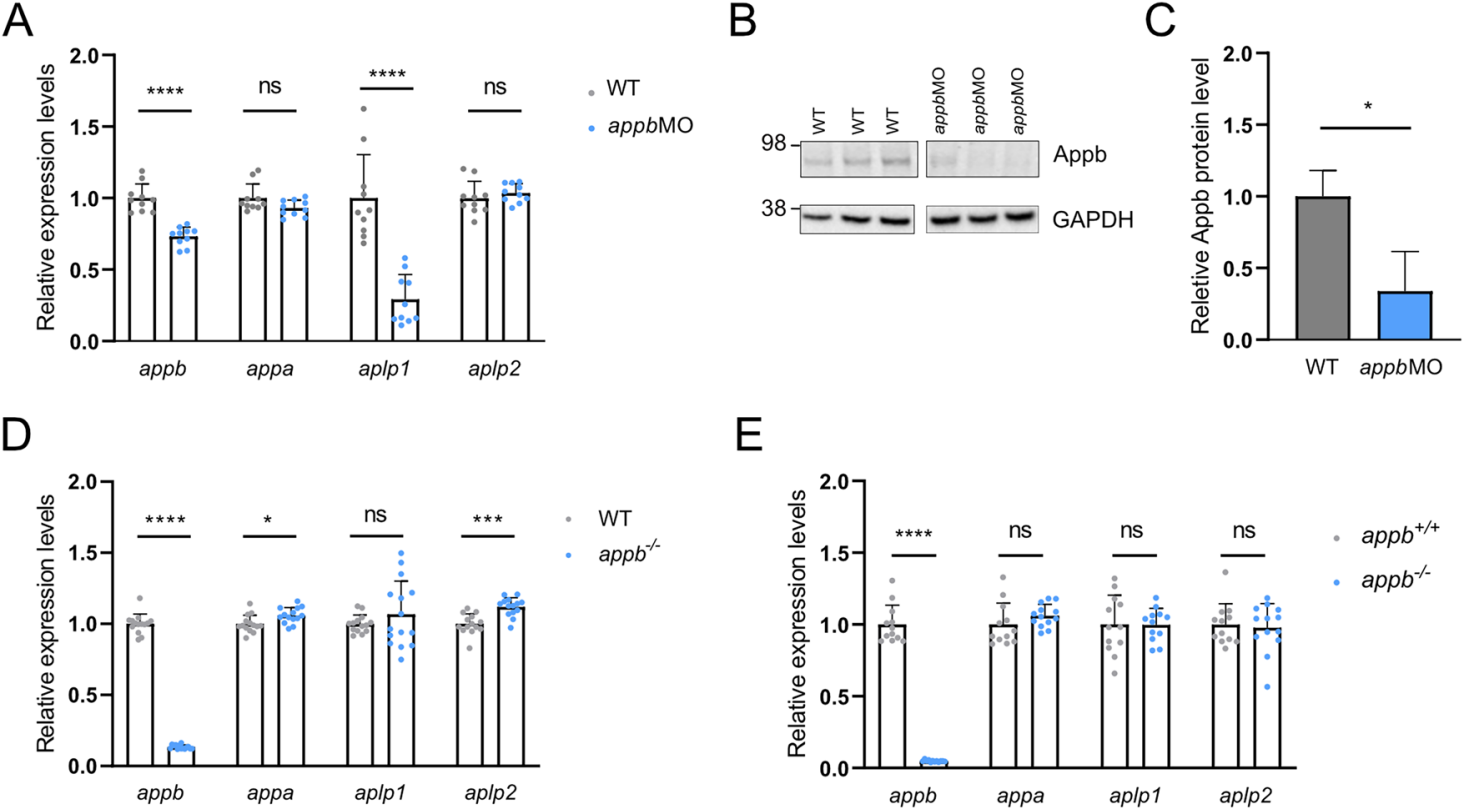
Relative gene expression in translation-blocking *appb*MO and *appb^−/−^*. ***A***, Relative expression level of *appa*, *appb*, *aplp2*, and *aplp1* (*N* = 10) in morphants compared with wild type (WT; *N* = 10) at 24 hpf. ***B***, Western blot analysis of Appb and GAPDH levels in wild type and translation-blocking *appb*MO at 3 dpf. ***C***, Quantification of Western blot data. ***D***, Relative expression of *appa*, *appb*, *aplp1*, and *aplp2* at 24 hpf in genetic *appb^−/−^* (*N* = 14) compared with WT (*N* = 14). ***E***, Relative expression of *appa*, *appb*, *aplp1*, and *aplp2* in adult brain of genetic *appb^−/−^* mutants (*N* = 13) and wild-type siblings (*N* = 12). Wild-type expression levels were set at 1. Data shown as mean + SD. ***A–E***, *n* = 3 biologically independent samples. Student's two-tailed *t* test was used to calculate *p* values. *p < 0.05, **p < 0.01, ***p < 0.005, and ****p < 0.001. Additional data relating to these analyses are provided in Extended Data Figure 2-1.

The above results, together with the observed degradation of mRNA transcript in *appb^−/−^* zebrafish (Banote et al., 2020), made us ask if the retained MC in *appb^−/−^* was the consequence of genetic compensation (Rossi et al., 2015; El-Brolosy et al., 2019; Ma et al., 2019) by other *app* family members. We therefore analyzed the mRNA levels of all *app* family members (*appa*, *appb*, *aplp1*, and *aplp2*) in both *appbMO*-injected wild-type (Fig. 2*A* and Extended Data Fig. 2-1*A*) and *appb^−/−^* larvae (Fig. 2*D*). Downregulation of *appb*, using either the splice-blocking morpholino or a translation-blocking morpholino, reduced *appb* mRNA and protein levels (Fig. 2*A–C* and Extended Data Fig. 2-1*A–C*) and *aplp1* mRNA levels but no change was observed on *appa* or *aplp2* expression levels (Fig. 2*A* and Extended Data Fig. 2-1*A*). While *appb mRNA* levels were reduced in *appb^−/−^* larvae, we observed a small but significant increase in the mRNA levels of *appa* and *aplp2* and although not significant, a trend toward upregulation of *aplp1* compared with their wild-type siblings (Fig. 2*D*). This suggests that while *appb* knockdown only affects *aplp1* expression, the genetic loss of function mutation in *appb* increase the mRNA levels of the more similar *appa* and *aplp2* genes. To evaluate if such compensation persists over time, we analyzed mRNA expression levels in the *appb^−/−^* adult brain. While *appb* expression level was still low, there was no significant change in the other *app* family members suggesting that TA may be more active during early stages of development (Fig. 2*E*). Together, these data show that a genetic *appb* mutation, but not a morpholino-mediated *appb* knockdown, upregulated transcript levels of *appa* and *aplp2* during early stages of development.

10.1523/ENEURO.0034-24.2024.f2-1 Figure 2-1 Relative gene expression and protein level in splice blocking *appb*MO. *A,* relative expression level of *appa*, *appb*, *aplp2* and *aplp1* (N *= *13) in *appb*MO compared with wildtype (N = 13) at 24 hpf. *B*, western blot analysis of Appb and GAPDH levels in wildtype and *appb*MO at 3dpf. *E,* quantification of western blot data. Wildtype expression levels were set at 1. Data shown as mean + SD. *A-C,* n = 3 biologically independent samples. Student’s two-tailed *t*-test was used to calculate *P* values. *P *< 0.05 (*), < 0.01 (**), < 0.005 (***) and *P *< 0.001 (****). Download Figure 2-1, TIF file.

### TA depends on *appb* mRNA degradation and not on protein level

**Figure 3.**
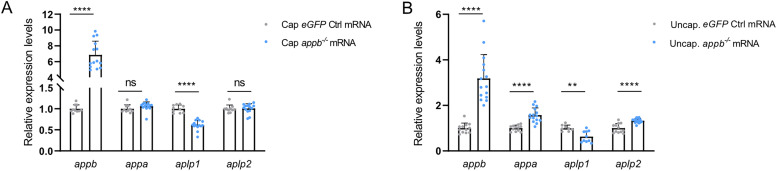
Expression of *app* family genes in embryos injected with unstable or stable *appb^−/−^* mRNA. ***A***, Injection of capped *appb^−/−^* RNA increases mRNA levels of *appb* but not mRNA level of *appa*, *aplp1*, or *aplp2* compared with control *eGFP* RNA (*N* = 10–14). ***B***, Injection of uncapped *appb^−/−^* RNA increases mRNA levels of *appb* and *appa*, compared with uncapped control *eGFP* RNA (*N* = 8–15). ***A***, ***B*** *n* = 3 biologically independent samples. Student's two-tailed *t* test was used to calculate *p* values. Wild-type expression levels were set to 1. Data shown as mean + SD. **p < 0.01 and ****p < 0.0001. ns, nonsignificant.

To investigate if the transcriptional response observed in *appb^−/−^* is activated by the presence of the PTC-containing *appb* mRNA, we injected stable, capped *appb^−/−^* mRNA into wild-type zebrafish. The use of mutant mRNA prevents translation of a functional protein from the injected mRNA and should thus not change the Appb protein level. Injection of capped *appb^−/−^* RNA, carrying a m^7^G(5′)ppp(5′)G at the 5′-end to protect the mRNA from degradation (Furuichi and Shatkin, 1977; Hsu and Stevens, 1993; Sachs, 1993), decreased *aplp1* mRNA levels but did not change *appa* or *aplp2* expression compared with the *eGFP* mRNA control (Fig. 3*A*). To test the effect of RNA decay, we used in vitro transcribed uncapped mutant *appb* mRNA, known to rapidly degrade due to 5′- to 3′ exonucleases (Mukherjee et al., 2012). Injection of uncapped mutant *appb* mRNA into wild-type zebrafish upregulated *appa* and *aplp2* and downregulated *aplp1* transcript levels at 24 hpf, compared with uncapped *eGFP* control mRNA (Fig. 3*B*). Together, this shows that *appb* mRNA decay, but not the mutation or the Appb protein level, upregulate *appa* and *aplp2* transcription, indicating a similar mechanism as described in TA (El-Brolosy et al., 2019). In contrast, the *aplp1* expression suggests a different response mechanism that does not depend on the specific mutation or mRNA degradation alone.

### No TA in an RNA-less *appb* mutant allele

**Figure 4.**
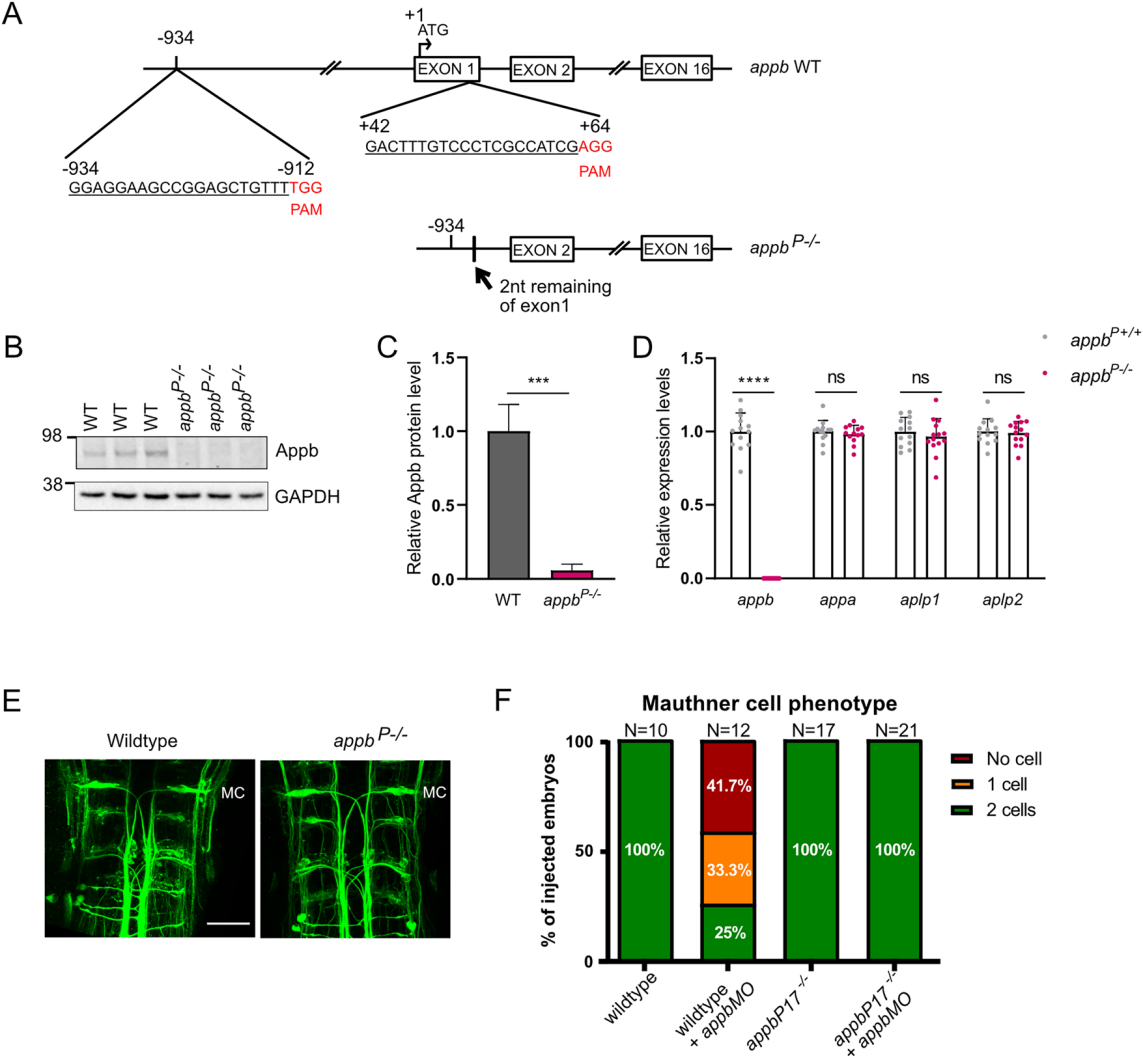
The RNA-less *appb^P^* mutation does not induce TA of other App family members. ***A***, Deletion of exon 1 and the 5′ upstream sequence of *appb* was performed using two gRNAs binding −934 bp 5′ to and 64 bp 3′ of the ATG start, where ***A*** is considered as +1. gRNA target sequences are underlined, and PAM sequences are marked in red. ***B***, Western blot analysis of Appb and GAPDH levels in wild type and *appb^P−/−^* at 3 dpf. ***C***, Quantification of Western blot data. ***D***, Relative expression levels of *appa* and *appb* (and Extended Data Fig. 4-1), *aplp1*, and *aplp2* in *appb^P−/−^* (*N* = 15) and wild-type *appbP^+/+^* siblings (*N* = 14). Ct values of *appb* in *appb^P−/−^* were above the detection threshold set to 40. ***E***, RMO44 staining in hindbrain of wild-type and *appb^P−/−^* mutants at 48 hpf. ***F***, Quantification of MC number in *appbMO* injected and noninjected wild type (WT) and *appb^P−/−^*. “*N*” indicates number of brains. MC, Mauthner cell. Scale bar, 50 μm. ***B***, ***D***, *n* = 3 biologically independent samples. ***F***, *n* = 2–3 biologically independent samples. Wild-type expression levels were set at 1. Data shown as mean + SD. Student's two-tailed *t* test was used to calculate *p* values. ***p < 0.005 and ****p < 0.0001. ns, nonsignificant. Additional data relating to these analyses are provided in Extended Data Tables 4-1–4-3.

Our results indicated that TA of *appa* and *aplp2* depends on mutant *appb* mRNA degradation. To test this hypothesis, we deleted the upstream 5′-region and transcription starts in *appb* exon 1 to generate a mutant without mRNA (shown as *appb^P−/−^* in this study). To this end, we used the CRISPR/Cas9 system to delete the 5′-upstream region and exon 1 of *appb* (Fig. 4). Two (gRNA89 and gRNA1064) out of 12 gRNAs (Extended Data Table 4-1) were efficient and used to remove a 972 bp fragment, including most of exon 1 (2 base pairs remaining) and its immediate 5′-upstream region (Fig. 4*A*). The generated *appb^P^* heterozygous mutants (*appb^P+/−^*) were outcrossed at least two generations and then inbreed to produce *appb^P^* homozygous mutants (*appb^P−/−^*). Western blot and qPCR confirm that the *appb^P^* homozygous mutants have very low or no Appb protein and *appb* mRNA production (Fig. 4*B*,*C*; Extended Data Fig. 4-1). Interestingly, the *appb^P−/−^* showed no change in *appa*, *aplp1*, or *aplp2* gene expression (Fig. 4*D*). However, neurofilament immunostaining showed that *appb^P−/−^* larvae, similarly to *appb* mutants, exhibit two bilaterally positioned M-cells (Fig. 4*E*) that are maintained after *appbMO* injections (Fig. 4*F*). This shows that M-cell development occurs in the absence of *appb* and suggests the involvement of mechanisms beyond TA.

10.1523/ENEURO.0034-24.2024.f4-1 Figure 4-1 Relative expression level of *appb* in *appb^P-/-^* compared to *appb^P+/+^*. Relative expression level of *appb* in *appb^P-/-^* at 24hpf (N = 12) and wildtype control (N = 13) with different assays binding different exons on *appb*. Wildtype mRNA levels were set at 1. n = 3 biological repeats. Data shown as mean + SD. Student’s two-tailed *t*-test was used to calculate *P* values. *P *< 0.001 (****). Download Figure 4-1, TIF file.

10.1523/ENEURO.0034-24.2024.t4-1 Table 4-1 List of gRNAs used to generate the appb^P-/-^ mutant. *Commoners denote modifications added to increase in vitro transcription yield by T7 polymerase*. Download Table 4-1, XLS file.

10.1523/ENEURO.0034-24.2024.t4-2 Table 4-2 Primers used for genotyping and Sanger sequencing. Download Table 4-2, XLS file.

10.1523/ENEURO.0034-24.2024.t4-3 Table 4-3 *Primers used to show the deletion of* appb *in* appb^P-/-^. Download Table 4-3, XLS file.

### Nonsense-mediated mRNA decay

**Figure 5.**
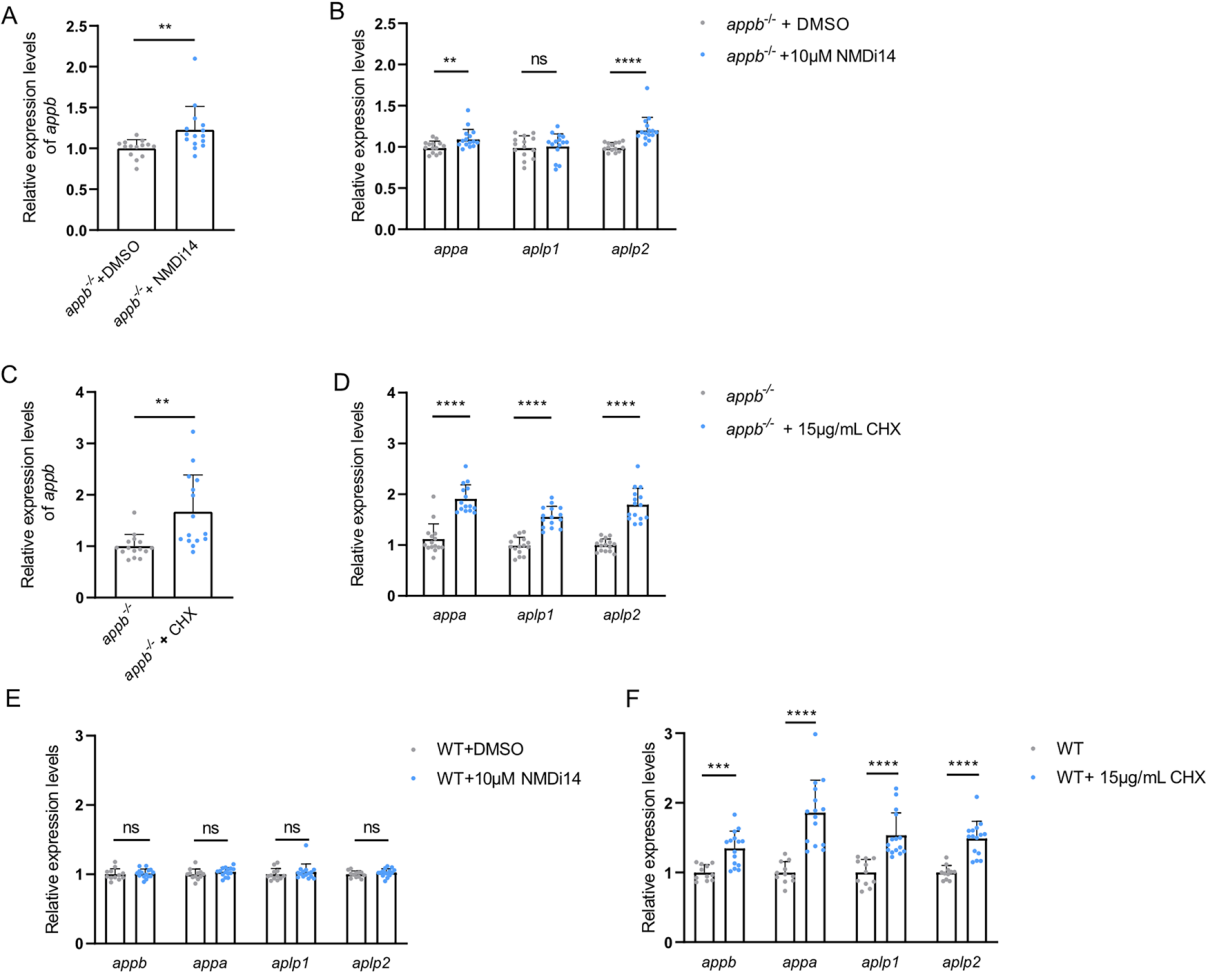
Expression of *app* family genes *appb^−/−^* and wild-type (WT) embryos after inhibition of NMD or translation and when knocking down of NMD core factors. ***A–D***, Relative mRNA expression levels of *appb*, *appa*, *aplp1*, and *aplp2* in *appb^−/−^* embryos treated with 0.1% DMSO or 10 μM NMDi14 between 24 and 48 hpf (*N* = 15) to inhibit nonsense-mediated mRNA decay (***A***,***B***) or with or without 15 μg/ml CHX between 24 and 29 hpf (*N* = 15) to block translation (***C***,***D***). ***E***, ***F***, Relative mRNA expression levels of *appb*, *appa*, *aplp1*, and *aplp2* in wild-type (WT) embryos treated with 0.1% DMSO or 10 μM NMDi14 between 24 and 48 hpf (*N* = 15) (***E***) or with or without 15 μg/ml CHX between 24 and 29 hpf (*N* = 15) (***F***). ***A–F***, *n* = 3 biologically independent samples. Data are shown as mean + SD. Student's two-tailed *t* test were used to calculate *p* values. *p < 0.05, **p < 0.01, ***p < 0.005, and ****p < 0.0001. ns, nonsignificant.

PTC containing mRNA generally activates NMD (Czaplinski et al., 1998; Kurosaki et al., 2019). To test the involvement of NMD in *appb^−/−^* mRNA decay, we pharmacologically blocked NMD using NMDi14, disrupting the UPF1–SMG7 interaction, or by blocking translation elongation with CHX, also known as a potent NMD inhibitor (Carter et al., 1995). Our data show a significant increase in *appb* mRNA levels in both NMDi14- and CHX-treated embryos (Fig. 5*A*,*C*). Furthermore, inhibition of NMD increased both *appa* and *aplp2* mRNA levels (Fig. 5*B*,*D*), with CHX treatment resulting in a more pronounced increase, also including *aplp1* mRNA levels. These results were contrary to the expected. Since decreased *appb* degradation would reduce TA, we predicted reduced *appa* and *aplp2* mRNA levels. Instead, our results suggest that while NMD is involved in *appb^−/−^* mRNA decay, the increased transcript levels indicate that the surveillance system regulates normal mRNA turnover of *app* family members. To test this, we inhibited NMD and found that CHX, but not NMDi14, led to a general increase of *appa*, *appb*, *aplp1*, and *aplp2* mRNA levels in wild-type larvae (Fig. 5*E*,*F*). Taken together, these data indicate a major role of NMD in the regulation of the physiological turnover of *appa*, *appb*, *aplp1*, and *aplp2* mRNAs.

**Figure 6.**
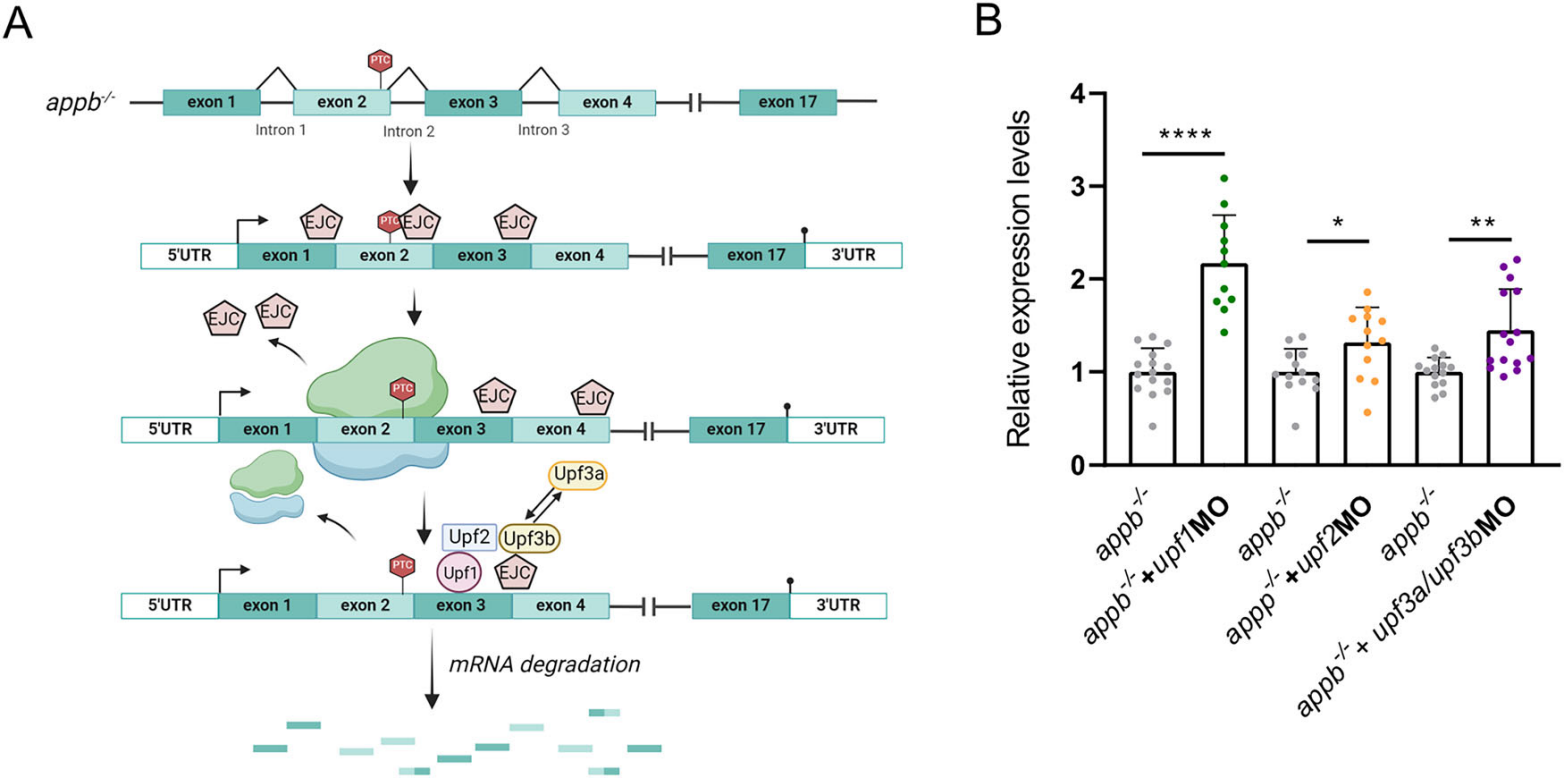
NMD pathway in *appb^−/−^*. ***A***, Illustration of NMD pathway activation in *appb^−/−^*. EJC, the exon-junction complex; PTC, premature termination codon. ***B***, Relative *appb* mRNA expression levels in *appb^−/−^* embryos injected with *upf1*MO (*N* = 12–15), *upf2*MO (*N* = 18–20), or *upf3a/upf3b*MO (*N* = 14–15) compared with uninjected *appb^−/−^*. *n* = 3 biologically independent samples. Data shown as mean + SD. Student's two-tailed *t* test were used to calculate *p* values. *p < 0.05, **p < 0.01, ***p < 0.005, and ****p < 0.0001. ns, nonsignificant. Additional data relating to these analyses are provided in Extended Data Figure 6-1.

Compensatory mechanisms induced by PTC-containing mRNA decay were recently suggested to be mediated through the NMD core factors, Upf1, Upf2, and Upf3b (El-Brolosy et al., 2019) or by a nondecay pathway involving Upf3a (Ma et al., 2019; Fig. 6*A*). To further investigate if these factors are involved in *appb^−/−^* mRNA decay, we knocked down the central proteins, Upf1, Upf2, Upf3a, and Upf3b, in *appb^−/−^*. If these proteins are involved in *appb* mRNA decay, then their downregulation should increase *appb* mRNA levels. Indeed, knockdown of all three core NMD factors increased *appb* mRNA levels in *appb^−/−^*, indicating their involvement in PTC-bearing *appb* mRNA decay (Fig. 6*B* and Extended Data Fig. 6-1).

10.1523/ENEURO.0034-24.2024.f6-1 Figure 6-1 Validation of morpholino knockdown of upf3b. A, the upf3bMO was designed to block the splicing region of exon3-intron3 of upf3b. PCR was performed with forward primer (FwdP) and reverse primer (RevP) to show the efficiency of injection of 1  ng upf3bMO. B, DNA fragment of 1233  bp contains 34  bp of exon 3, 336  bp of exon 4, 5, 6 and a part of exon 7 and the whole intron 3 of upf3b. Download Figure 6-1, TIF file.

**Figure 7.**
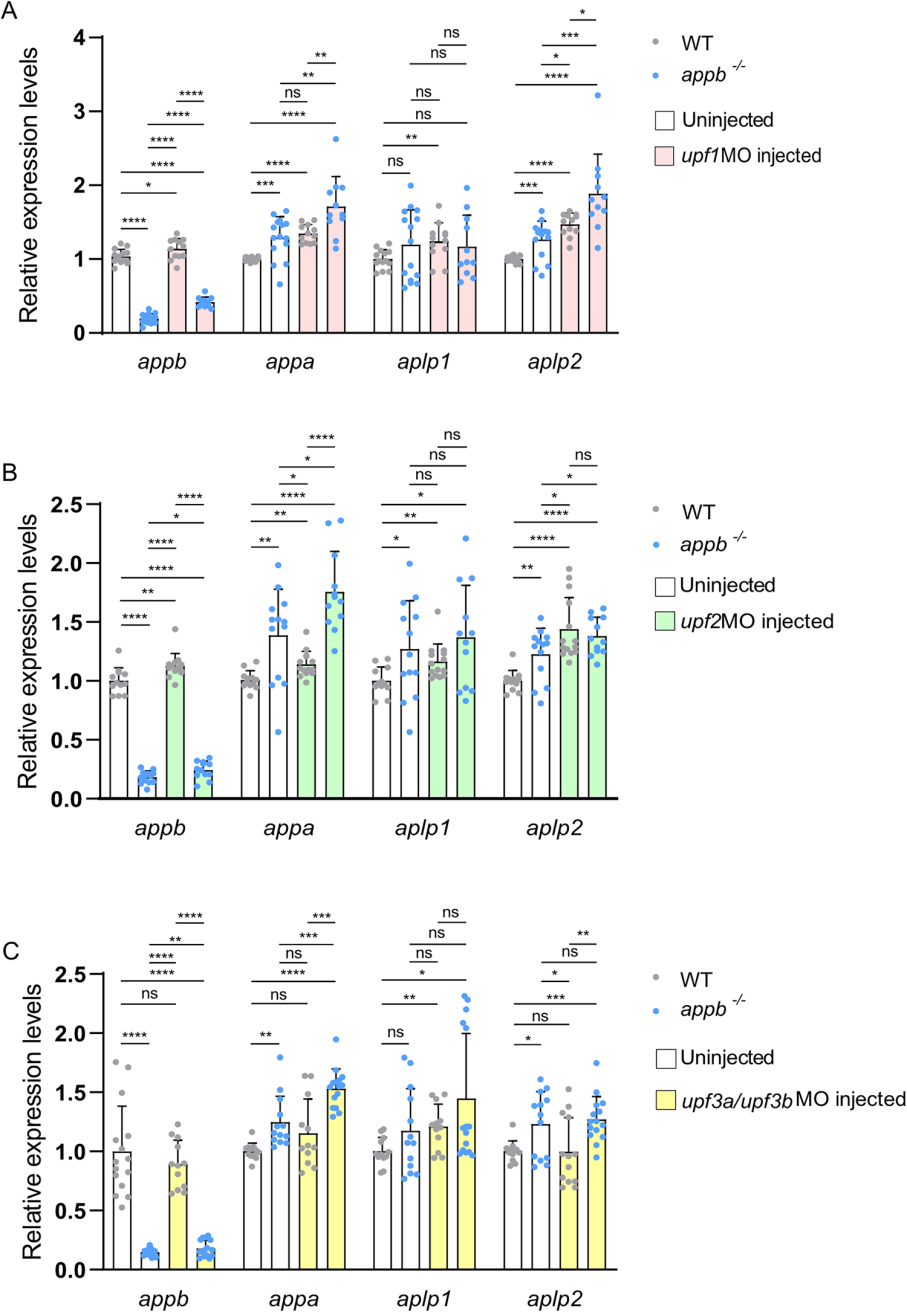
Expression of *app* family genes in wild-type and *appb^−/−^* embryos before and after knocking down NMD factors. ***A–C***, Relative mRNA expression levels of *appb*, *appa*, *aplp1*, and *aplp2* in uninjected wild-type embryos (*N* = 12–15), uninjected *appb^−/−^* (*N* = 13–15), wild type injected with *upf1*MO (*N* = 12), *upf2*MO (*N* = 14), *upf3a/upf3b*MO (*N* = 13), and *appb^−/−^* injected with *upf1*MO (*N* = 11), *upf2*MO (*N* = 12), and *upf3a/upf3b*MO (*N* = 15). *n* = 3 biologically independent samples. Data shown as mean + SD. Student's two-tailed *t* test were used to calculate *p* values. *p < 0.05, **p < 0.01, ***p < 0.005, and ****p < 0.0001. ns, nonsignificant.

To determine the role of NMD in TA and normal mRNA turnover, we downregulated *upf1*, *upf2*, and *upf3*s in both wild-type and *appb^−/−^* larvae and analyzed mRNA levels of all *app* family members (Fig. 7). Downregulation of *upf1* and *upf2* increased mRNA levels of *appa* and *aplp2* in wild-type and *appb^−/−^* larvae compared with the uninjected controls (Fig. 7*A*,*B*). Furthermore, NMD inhibition in *appb^−/−^* significantly increased expression of *appa* and *aplp2* compared with the injected controls with a similar change observed in *appa* expression after *upf2* inhibition. Thus, there is a gradual increase in *appa* and *aplp2* mRNA levels where downregulation of *upf1* or *upf2* in wild types led to a similar upregulation as detected in *appb^−/−^*, with even more elevated levels observed in injected *appb^−/−^* (Fig. 7*A*,*B*). In contrast, the mRNA levels of *aplp1* increased with *upf2* inhibition in both wild types and *appb^−/−^*, while the *aplp1* increase was only observed in wild types (not in *appb^−/−^*) with a *upf1* knockdown. It is however likely that there is a general increase in *aplp1* but that the sample variation obscured this effect (Fig. 7). Together, our data suggest that upregulation of *appa* and *aplp2*, when knocking down *upf1* and *upf2*, is due to both increased mRNA stability and TA due to the remaining *appb^−/−^* mRNA decay, since knockdown of *upf1* and *upf2* did not rescue a*ppb* mRNA decay in *appb^−/−^*.

Contrary, knockdown of *upf3a/upf3b* only changed the mRNA levels in *appb^−/−^* indicating that Upf3s mainly are active in the presence of a PTC (Fig. 7*C*). Knockdown of *upf3a/upf3b* increased *appb* mRNA levels in *appb^−/−^* and in addition to *appa* and *aplp2*, also increased *aplp1* mRNA levels compared with uninjected wild-type controls. These results indicate that while Upf3s mediate degradation of mutant *appb* mRNA, they are not involved in regulation of the normal mRNA turnover.

**Figure 8.**
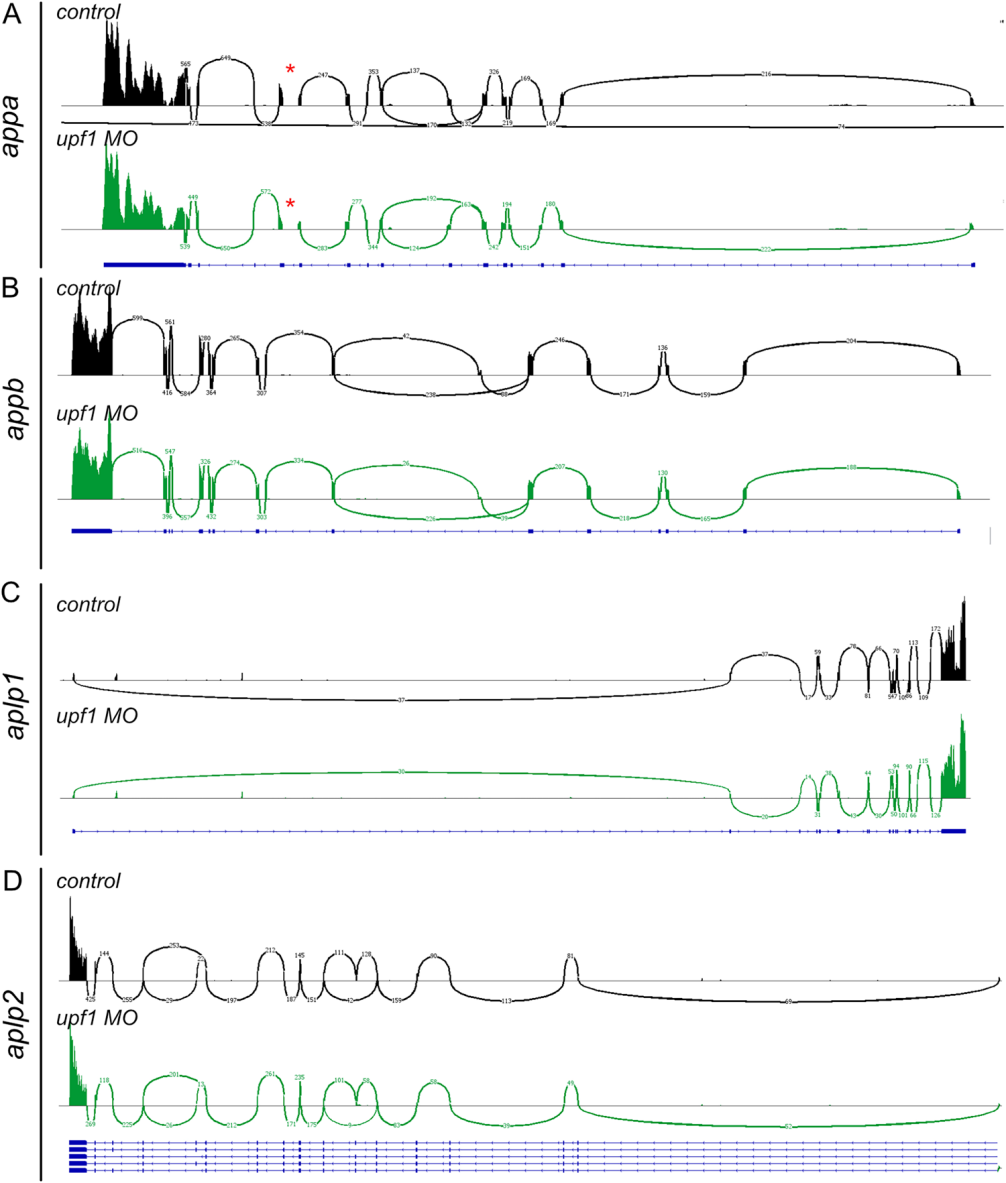
Sashimi plots of *app* family members in wild-type controls and *upf1* morpholino injected larvae at 24 hpf. ***A–D***, Splicing of *appa* (***A***), *appb* (***B***), *aplp1* (***C***), and *aplp2* (***D***) are shown in wild-type controls (black) and *upf1* morpholino injected (green) larvae. Numbers indicate number of RNAseq reads and reference gene is outlined in blue. Red star indicates lack of splice junction between exons in *appa*. Additional data relating to these analyses are provided in Extended Data Figure 8-1 and Extended Data Table 8-1.

The surveillance mechanism of the NMD pathway is preventing faulty mRNAs from being translated. An alternative explanation to the increased mRNA levels observed in wild types upon NMD inhibition would be the presence of alternative splice variants including poisonous exons or other faulty splice events. To determine if such events were present, we analyzed transcript variants present in wild-type and *Upf1* knockdowns by RNA sequencing. Sashimi plots of mRNA sequencing reads showed no alternative splicing of *appa*, *appb*, *aplp1*, and *aplp2* in *upf1* knockdown embryos compared with wild-type controls (Fig. 8*A–D*). Interestingly exon 11–12 in *appa* lacked splice arches (marked with red box in Fig. 8*A*) suggesting that no RNA reads spanned these exons. This region is differently annotated in Refseq and Ensembl, and by manual curation, we found that insertion of the contig LKPD02013461.1 between exons 11 and 12 gave a better sequence alignment that matched with de novo assembled RNAseq data (data not shown; illustrated in Extended Data Fig. 8-1). Furthermore, based on our data, evaluation of percent spliced-in (PSI) values did show a significantly increased number of alternative splicing of other genes in the *Upf1* knockdowns samples; however none of the *app* family members were included among those (Extended Data Table 8-1). These results suggest that the increased transcripts level observed after NMD inhibition corresponds to normal protein coding mRNA transcripts.

10.1523/ENEURO.0034-24.2024.f8-1 Figure 8-1 Outline of the gene assembly of the appa gene on Chromosome 1. The appa gene assembly in Ensembl (A*), Refseq (*B*) and our manually assembled (*C*) adding contig LKPD02013461.1 to a region with di-nucleotide repeats in contig FP067437.2. The inclusion of this sequence is supported by contig JALCZS010004005.1 which cover flanking regions. Numbers and dotted lines indicated positions on chromosome 1*. Download Figure 8-1, TIF file.

10.1523/ENEURO.0034-24.2024.t8-1 Table 8-1 *PSI-sigma data. Splice events in wildtype controls and* upf1*MO injected larvae at 24hpf*. Download Table 8-1, XLS file.

### TA in human neuronal progenitor cells but not in differentiated human neuronal cells in vitro

**Figure 9.**
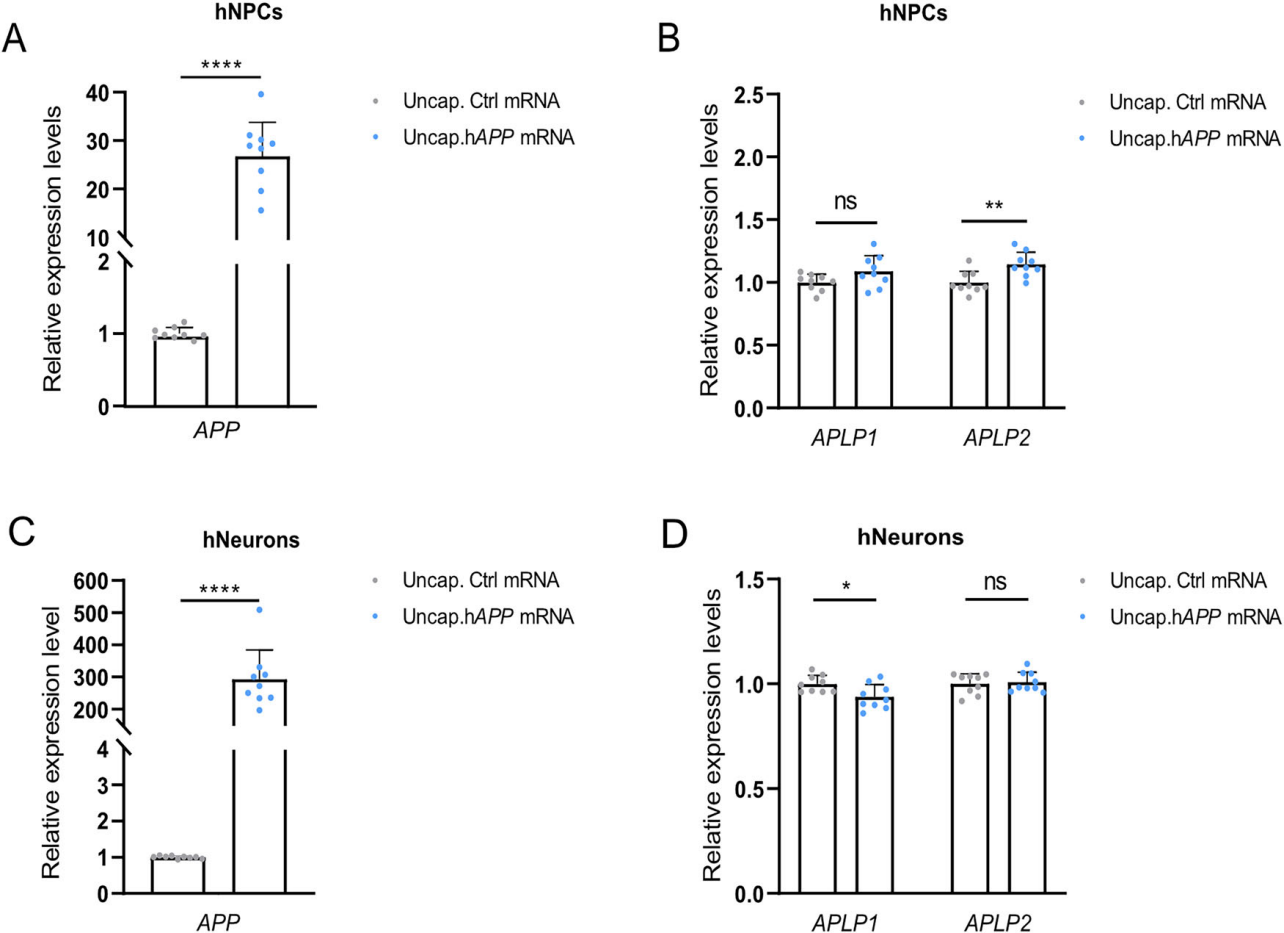
TA in hNPCs but not in terminally differentiated neuron cells transfected with unstable h*APP695* mRNA. ***A***, Relative *APP* level in hNPCs transfected with uncapped *eGFP* (control) or *hAPP695* mRNA (*N* = 9). ***B***, *APLP1* and *APLP2* levels in hNPCs transfected with uncapped *eGFP* (control) or h*APP695* mRNA (*N* = 9). ***C***, Relative *APP* level in human neuron cells transfected with uncapped *eGFP* or h*APP695* mRNA (*N* = 9). ***D***, Relative mRNA level of *APLP1* and *APLP2* in terminally differentiated neuron cells transfected with uncapped *eGFP* or h*APP695* mRNA (*N* = 13). *APP* in control *eGFP* transfected cells were set at 1. ***A–D***, *n* = 3 biologically independent samples. Data are mean + SD. Student's *t* test was used to calculate *p* values. *p < 0.05, **p < 0.01, ***p < 0.005, and ****p < 0.0001. ns, nonsignificant.

To address if the compensation found in zebrafish could be translated to human neurons, we used human neuronal progenitor cells (hNPCs) and neurons differentiated from hiPSCs. Transfection of both cell types with uncapped human *APP* (h*APP*) mRNA resulted in increased *APP* levels (Fig. 9*A*,*C*). However, upregulation of *APLP2* was only observed in multipotent hNPCs (Fig. 9*B*) and not in terminally differentiated neurons (Fig. 9*D*). No change was observed in *APLP1* expression (Fig. 9*B*,*D*). This suggests that the TA response is lower in terminally differentiated neurons compared with neuronal progenitors.

## Discussion

**Figure 10.**
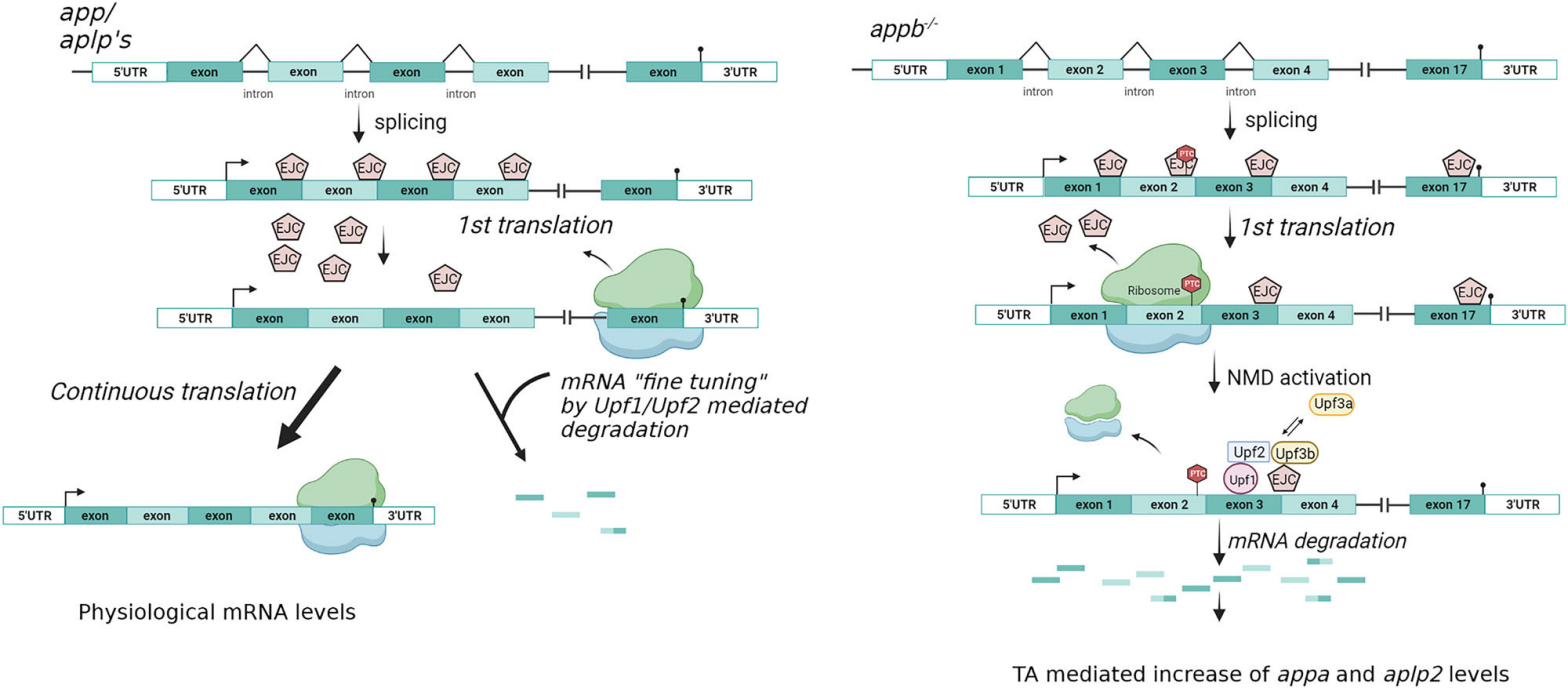
NMD pathway. ***A***, ***B***, Illustration of the mediated mRNA regulation of *app* family members under physiological conditions (***A***) and in *appb* mutants (***B***).

Genetic approaches aiming to understand the function of genes or mutations involved in disease frequently confront the absence of phenotypes. In zebrafish, the mismatch commonly observed between morpholino knockdown and genetic mutations further confound the question on how the techniques used to introduce genetic modifications affect the phenotypic outcome (Kok et al., 2015). Thus, understanding the underlying mechanisms would not only facilitate genetic approaches for functional gene studies but could also serve in the search for disease-modifying therapies. The partly unclear physiological functions of APP and the APLPs have been attributed to redundancy between family members (reviewed in Muller et al., 2017). Although APP and APLP2 functionally can substitute for each other, the question regarding if and how the loss of one gene activates the expression of other homologous genes remains. Here we show that mutations in the *appb* gene in zebrafish induce TA of other family members through mRNA degradation. Furthermore, our data shows that the NMD surveillance pathway regulate the physiological transcript levels of all *app* family members (Fig. 10).

The involvement of NMD in regulation of physiological transcript levels has gained increasing interest and is now known to be an important part of biological processes such as spermatogenesis, cell stress, development, and neurogenesis (reviewed in Nasif et al., 2018). Thus, impairments in NMD proteins are associated with intellectual disability but also seem to be linked to neurodegenerative disorders including amyotrophic lateral sclerosis (ALS; Z. Sun et al., 2011; Jackson et al., 2015). Here we found that the expression of *app* family genes is regulated by the NMD pathway, and our data suggest Upf1 and Upf2 to be central factors in both wild type and mutants. Contrary, Upf3 only seems to be active in PTC-carrying mutants, which is in accordance with previous reports suggesting that Upf3 predominantly triggers NMD as an EJC in PTC-carrying mRNAs (Nguyen et al., 2012, 2014; Bushman et al., 2015; Yi et al., 2021). In contrast to a previous report showing changed pre-mRNA splicing reported in a genetic *upf1* mutant (Lawir et al., 2020), our data support a role of the NMD system in regulating the level of normal protein coding mRNA transcripts. Thus, we propose here that the NMD pathway act to fine-tune the physiological expression level of members of the App family.

Through its function in scavenging faulty mRNAs, the NMD system is also part of activating TA, either through mRNA degradation via Upf1, Upf2, and Upf3b or through the GCR-activating complex in which Upf3a and Wdr5 act to upregulated homologous genes without mRNA decay (Ma et al., 2019). Our data suggest that activation of TA by lesions in an *app*-gene does not depend on protein level but mRNA degradation since injection of a mutant transcript in wild types but not the RNA-less mutants changed transcription levels of other homologs. However, it is important to note that all lesions do not induce the same response. Here, downregulation of *appb* using either the PTC introducing splice-blocking *appb* morpholino (Abramsson et al., 2013) or the translation-blocking morpholino both decreased *appb* mRNA and protein levels but did not change transcript levels of *appa* and *aplp2* compared with controls. These findings are in accordance with a previous study by El-Brolosy et al., suggesting that some lesions result in strong NMD while others do not, mainly due differences in mRNA decay (El-Brolosy et al., 2019). Similarly, here the difference observed in TA could be due to the stronger *appb* mRNA decay in the genetic *appb* mutant compared with the splice blocking morpholino. The remaining mRNA decay could also explain why TA was still detected in mutants with blocked NMD. The mechanisms by which Upf1 mediates mRNA decay depends on the interacting proteins available, which is determined by the cells’ proliferative and differentiation state (Z. Sun et al., 2011; L. Sun et al., 2023). Our data indicate that Upf1–SMG7 interaction is present in PTC-containing mutants but that the interaction is not required for physiological *app* family expression regulation. The moderate increase in PTC-containing *appb* mRNA level after both genetic and pharmacological inhibitions of the NMD pathway indicate that knockdown of NMD is not enough to fully block *appb* mRNA decay. Furthermore, the regulatory function of NMD on normal transcript levels complicated our analysis, since inhibition of the NMD system led to elevated transcript levels in the absence of mutations and even higher levels in the presence of a PTC-carrying mutation. Taken together, the increased transcript level in NMD-inhibited *appb^−/−^* is therefore likely an accumulated effect composed of an increase in normal transcript and TA driven by the remaining *appb* mRNA decay.

Furthermore, we noted that the *app* family genes responded slightly different to the *appb^−/−^* mutation. While *appb* mRNA degradation increased *appa* and *aplp2* expression, the often-inverse response of *aplp1* indicates a potentially different regulatory mechanism. Previous reports have suggested that TA is most active between genes with a high degree of conservation. The nucleotide sequence of *aplp1* is more distantly related with *appb* compared with *appa* and *aplp2* which may be one reason for the observed response. We also note that downregulation of *aplp1* correlates with the presence of wild-type *appb* mRNA while upregulation is observed in the absence of wild-type *appb* mRNA. It is therefore plausible that the *aplp1* mRNA level is linked to Appb protein levels. Finally, while we have shown TA between *app* family members, the lack of Mauthner cell phenotype in the mRNA-less mutants argues for additional mechanisms, used to sense loss of App family members. While the mRNA defect introduced by the splice-blocking morpholino is present in both wild types and *appb^−/−^*, the influence of a faulty mRNA is absent in mRNA-less mutants. Different mutations have varying effect on the transcriptome, but the extent to which such variations affect cell fate and differentiation remains to be analyzed. This furthermore shed light on the complex regulation of this protein family.

Although the presence of TA in early development is clear, our data indicate that this mechanism may be restricted to nascent neurons or neuronal progenitors as we could not detect any changes in transcript level in adult zebrafish brain or in terminally differentiated human neuronal cells cultured in vitro. This is consistent with results indicating that part of the NMD proteins are downregulated during differentiation of neuronal stem cells (Alrahbeni et al., 2015) and that the activity of the NMD machinery differs between cell types (Zetoune et al., 2008). In addition, it is possible that a strong TA activation in less differentiated cells is overshadowed by the nonresponding cells. That would also explain why analysis of *App*, *Aplp1*, and *Aplp2* single mutant mice did not support elevated expression of other family members, neither on protein (von Koch et al., 1997) nor on mRNA level (Aydin et al., 2011). Nevertheless, it is likely that TA may complicate functional studies of *APP* and *APLPs* when using genetic mutations that enhance mRNA decay.

The involvement of TA in disease is a challenging question. In humans, APP dosage is critical in the pathogenesis of Alzheimer's disease (AD) as individuals carrying three copies of *APP* due to a partial or complete duplication of chromosome 21 (Down syndrome) relentlessly develop AD (Zigman et al., 2002; McCarron et al., 2014). In sporadic AD, reports on increased APP levels also suggest that the expression correlates with amyloid levels and may be crucial for the disease progression (Beyreuther et al., 1993; Preece et al., 2004; Matsui et al., 2007). Thus, based on our study, genetic alterations in APLP2 or genes in the NMD pathway could contribute to changed APP levels in the adult brain. Such events would inevitably promote amyloid formation and AD progression.

In summary, we describe a mechanism by which mutations in one App family member activate TA to induce the expression of other App family members to compensate for its loss and that this mechanism is present both in zebrafish and human NPC. Such compensation is, according to our data, an early event in neurogenesis as TA only was observed in neuronal progenitor cells and not in the adult brain or in vitro cultured human terminally differentiated neuronal cells. We also propose that the NMD pathway is involved in regulating the physiological transcript levels of all App family members. However, the extent to which the NMD-mediated regulation of APP family members contribute to brain homeostasis and disease remains to be investigated.

## Synthesis

Reviewing Editor: Deanna Smith, University of South Carolina Decisions are customarily a result of the Reviewing Editor and the peer reviewers coming together and discussing their recommendations until a consensus is reached. When revisions are invited, a fact-based synthesis statement explaining their decision and outlining what is needed to prepare a revision will be listed below. The following reviewer(s) agreed to reveal their identity: Lindsay Rizzardi. Note: If this manuscript was transferred from JNeurosci and a decision was made to accept the manuscript without peer review, a brief statement to this effect will instead be what is listed below.

The reviewers and I agree that the manuscript has improved and most of the concerns have been addressed. The manuscript convincingly shows that NMD regulates basic levels of app genes in WT conditions, but the arguments for TA need better justification. Please address the following concerns:

1) The splice modulating MO used leads to a PTC that is expected to elicit NMD (Abramsson et al, Dev biol, 2013). The authors need to comment on why that did not elicit TA. In the 2019 study from the Stainier lab, they showed that stronger levels of NMD elicit better TA, the authors may want to comment on whether appb -/- elicited an upregulation of appa or aplp2 because they seemingly have better NMD, and thus TA". The paper will also benefit from using a translation blocking MO rather than a splice modulating ASO as a control.

2) Fig. 3A: refrain from saying the injected mRNA is a PTC mRNA as when injecting mature mRNA, they lack the deposited EJC proteins during splicing and thus the PTC won't be recognized as premature stop but rather a "regular" stop.

3) The authors should provide better discussion on why the promoterless appb fish did not show a phenotype. One issue with promoterless allele is they are often not 100% null and residual transcription still exist. While the authors do show by qPCR that the transcript levels are absent, that is "relative" qPCR, and the Ct values obtained from the qPCR experiment should be shown in the figure for reference. What may support that the promoterless is not 100% efficient in blocking transcription is the 20-25% residual protein being produced from the mutant as obvious in Fig. 4C. There is a chance those 25% are enough, and it would help to compare the protein levels in those mutants relative to the knockdown animals in terms of understanding the different observed phenotypes despite lack of compensation in both.

4) In Figures 5 and 6, it is obvious that app genes are regulated by NMD. Testing if NMD is required for the upregulations of appb and aplp2 (and thus if it is TA) may become challenging as a) if TA is involved, then loss of NMD should decrease appb and aplp2 transcription through lack of TA (i.e. decrease premRNA production), but at the same time b) the transcripts by themselves in WT fish are stabilized by loss of NMD (i.e. the mature mRNA is less degraded), leading to opposing effects and an apparent false negative result that potential TA-induced appa or aplp2 upregulations in appb -/- fish are not affected by loss of NMD. To overcome this complication, the authors should either 1) assess appa and aplp2 premRNA levels when blocking NMD genetically or pharmacologically in the mutant animals, or 2) compare WT fish where NMD was inhibited to mutant fish where NMD was also inhibited, report appa and aplp2 levels in such comparison relative to WT and mutant fish where NMD was not inhibited as was performed in the 2019 Stainier lab study.

Minor comment:

The statistical test used in Fig. 3 is not mentioned in the legend.

## Author Response

Response to reviewer comments Synthesis Statement for Author (Required):

The reviewers and I agree that the manuscript has improved and most of the concerns have been addressed. The manuscript convincingly shows that NMD regulates basic levels of app genes in WT conditions, but the arguments for TA need better justification. Please address the following concerns:

1) The splice modulating MO used leads to a PTC that is expected to elicit NMD (Abramsson et al, Dev biol, 2013). The authors need to comment on why that did not elicit TA. In the 2019 study from the Stainier lab, they showed that stronger levels of NMD elicit better TA, the authors may want to comment on whether appb -/- elicited an upregulation of appa or aplp2 because they seemingly have better NMD, and thus TA". The paper will also benefit from using a translation blocking MO rather than a splice modulating ASO as a control.

Response: We strongly agree with this comment and thank the reviewer for the thorough thoughts on our work. We have now commented on the fact that the splice blocking appbMO, while still introducing a PTC, do not activate TA (Line 535-567).

In addition, we show that appb knockdown using a translation blocking MO (line 377-381,535-44, and Figure 2 ) do not change mRNA expression levels similar to the splice blocking morpholino.

2) Fig. 3A: refrain from saying the injected mRNA is a PTC mRNA as when injecting mature mRNA, they lack the deposited EJC proteins during splicing and thus the PTC won't be recognized as premature stop but rather a "regular" stop.

Response: We have taken notice of this and have changed the use of PTC accordingly (Line 404 and 408).

3) The authors should provide better discussion on why the promoterless appb fish did not show a phenotype. One issue with promoterless allele is they are often not 100% null and residual transcription still exist. While the authors do show by qPCR that the transcript levels are absent, that is "relative" qPCR, and the Ct values obtained from the qPCR experiment should be shown in the figure for reference.

What may support that the promoterless is not 100% efficient in blocking transcription is the 20-25% residual protein being produced from the mutant as obvious in Fig. 4C. There is a chance those 25% are enough, and it would help to compare the protein levels in those mutants relative to the knockdown animals in terms of understanding the different observed phenotypes despite lack of compensation in both.

Response: We thank the reviewer for this specific and very relevant question. We have re-analyzed protein expression and found that with the new detection method the total Appb protein level detected became lower, yet not zero (line 240-248 in M&M and 420). Also, the primers used to detect appb mRNA are outside the mutation and can detect the transcript. We increased the Ct detection limit to 40 and could still not detect any signal (Figure 4, line 420).

The protein analysis is now exchanged with the new data. We therefore conclude that the appbP-/- produce very low, if any appb mRNA and Appb protein.

Furthermore, knockdown of appb using either a splice- or translation blocking MO decreased Appb protein levels to 40-50% of controls. Together this data support that the lack of phenotype in appbP-/- most likely is not due to remaining APP protein.

Finally, we have added an extended discussion on the normal Mauthner cell phenotype in promoter-less mutants in the discussion although we still do not know the exact mechanism (line 568-572).

4) In Figures 5 and 6, it is obvious that app genes are regulated by NMD. Testing if NMD is required for the upregulations of appb and aplp2 (and thus if it is TA) may become challenging as a) if TA is involved, then loss of NMD should decrease appb and aplp2 transcription through lack of TA (i.e. decrease pre-mRNA production), but at the same time b) the transcripts by themselves in WT fish are stabilized by loss of NMD (i.e. the mature mRNA is less degraded), leading to opposing effects and an apparent false negative result that potential TA-induced appa or aplp2 upregulations in appb -/- fish are not affected by loss of NMD.

To overcome this complication, the authors should either 1) assess appa and aplp2 premRNA levels when blocking NMD genetically or pharmacologically in the mutant animals, or 2) compare WT fish where NMD was inhibited to mutant fish where NMD was also inhibited, report appa and aplp2 levels in such comparison relative to WT and mutant fish where NMD was not inhibited as was performed in the 2019 Stainier lab study.

Response: We agree with the reviewer on the fact that it is challenging to know the transcriptional effect of NMD versus TA. We have addressed the question by remaking figure 6 into one figure with controls and appb mutants with or without blocking NMD (New figure 7). This also led to some rearrangements in figure 5 and 6.

When comparing mRNA levels of appa and aplp2 across the different samples it becomes clear that the Upf's act somewhat different in wildtypes and mutants. However, in many aspects the effect of the appb-/- mutation is similar with knockdown of upf's. We think that the new graphs, comparing both uininjected and Upf knockdown, contribute to a better understanding of the impact of TA and NMD relative to the control. We interpret the additional increase in appa and to a lesser extent aplp2 transcript level in appb-/- + upf knockdown to represents TA induced by the remaining appb mRNA decay (Line 451-470, 536-545).

Minor comment:

The statistical test used in Fig. 3 is not mentioned in the legend.

Response: We added the statistical test used in Figure 3 (line 910)
