# Supplementary figures and images for: Fine-Tuning Amyloid Precursor Protein Expression through Nonsense-Mediated mRNA Decay
Source: eNeuro. 2024 Jun 3;11(6):ENEURO.0034-24.2024. doi: 10.1523/ENEURO.0034-24.2024 (PMC11164851; doi:10.1523/ENEURO.0034-24.2024)

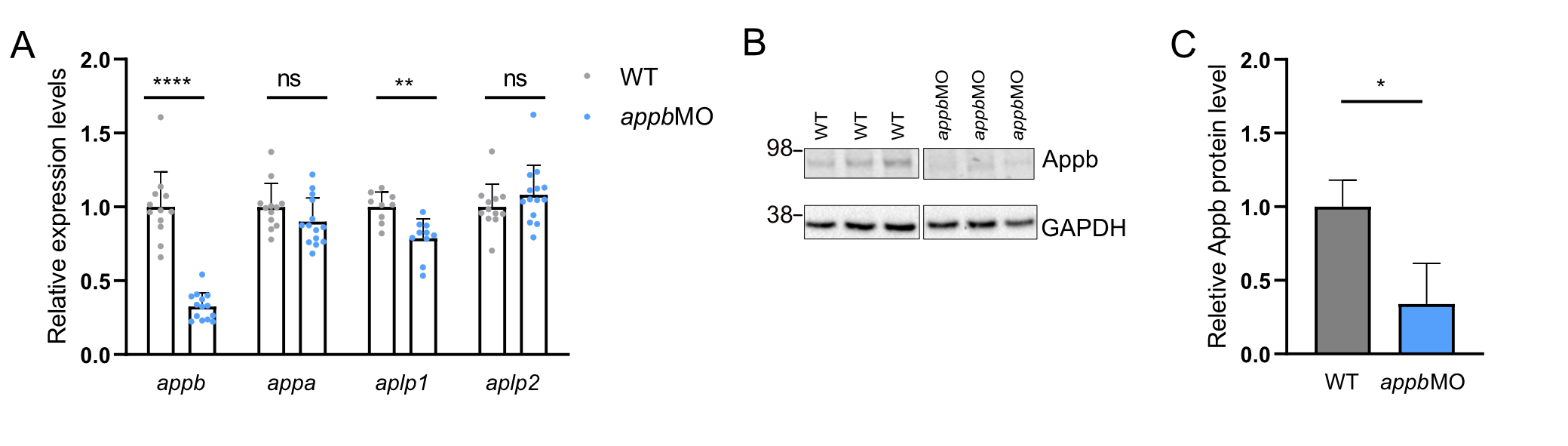

Supplement: Figure 2-1 — Relative gene expression and protein level in splice blocking appbMO. A, relative expression level of appa, appb, aplp2 and aplp1 (N = 13) in appbMO compared with wildtype (N = 13) at 24 hpf. B, western blot analysis of Appb and GAPDH levels in wildtype and appbMO at 3dpf. E, quantification of western blot data. Wildtype expression levels were set at 1. Data shown as mean + SD. A-C, n = 3 biologically independent samples. Student’s two-tailed t-test was used to calculate P values. P < 0.05 (*), < 0.01 (**), < 0.005 (***) and P < 0.001 (****). Download Figure 2-1, TIF file. [file eneuro-11-ENEURO.0034-24.2024-s002.tif]

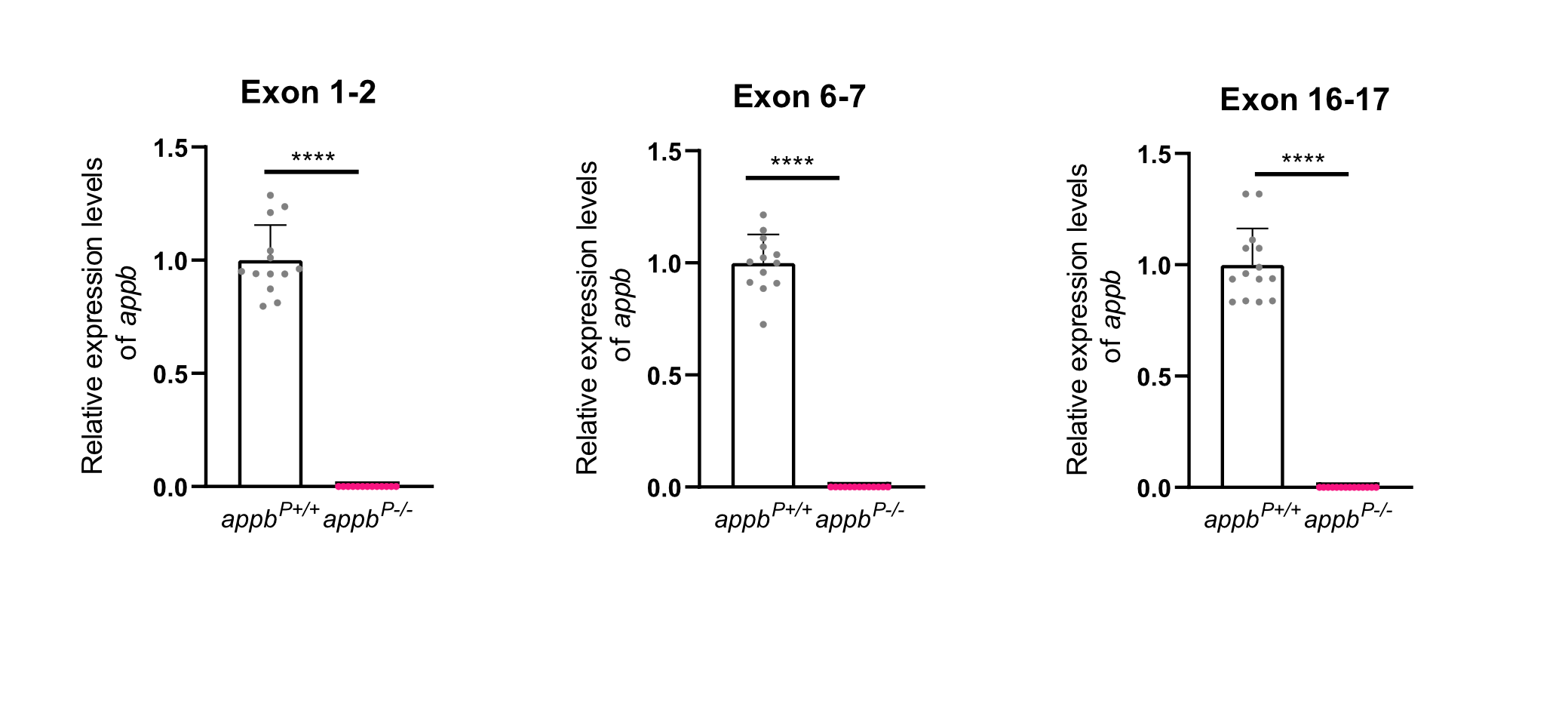

Supplement: Figure 4-1 — Relative expression level of appb in appbP-/- compared to appbP+/+. Relative expression level of appb in appbP-/- at 24hpf (N = 12) and wildtype control (N = 13) with different assays binding different exons on appb. Wildtype mRNA levels were set at 1. n = 3 biological repeats. Data shown as mean + SD. Student’s two-tailed t-test was used to calculate P values. P < 0.001 (****). Download Figure 4-1, TIF file. [file eneuro-11-ENEURO.0034-24.2024-s003.tif]

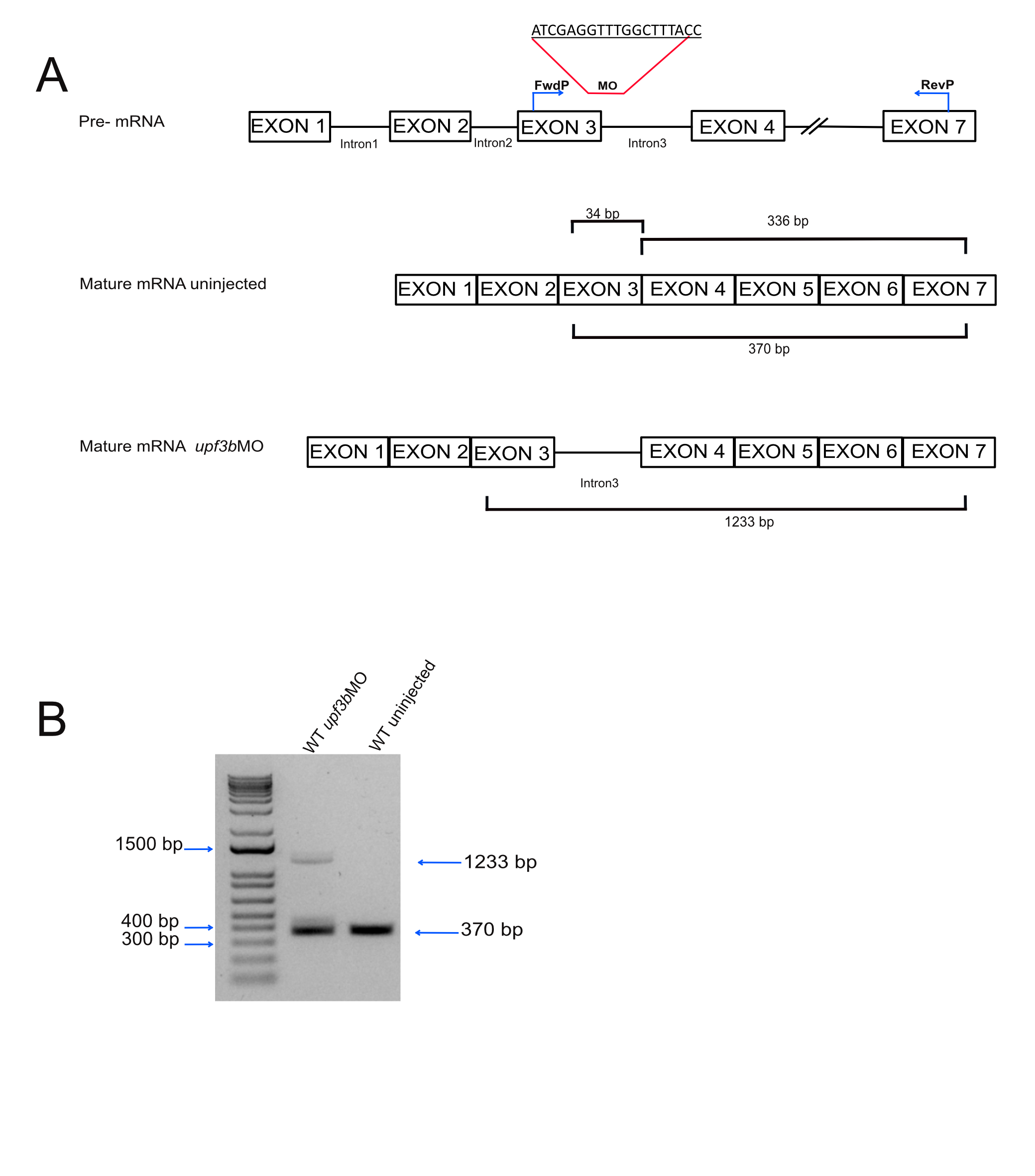

Supplement: Figure 6-1 — Validation of morpholino knockdown of upf3b. A, the upf3bMO was designed to block the splicing region of exon3-intron3 of upf3b. PCR was performed with forward primer (FwdP) and reverse primer (RevP) to show the efficiency of injection of 1 ng upf3bMO. B, DNA fragment of 1233 bp contains 34 bp of exon 3, 336 bp of exon 4, 5, 6 and a part of exon 7 and the whole intron 3 of upf3b. Download Figure 6-1, TIF file. [file eneuro-11-ENEURO.0034-24.2024-s004.tif]

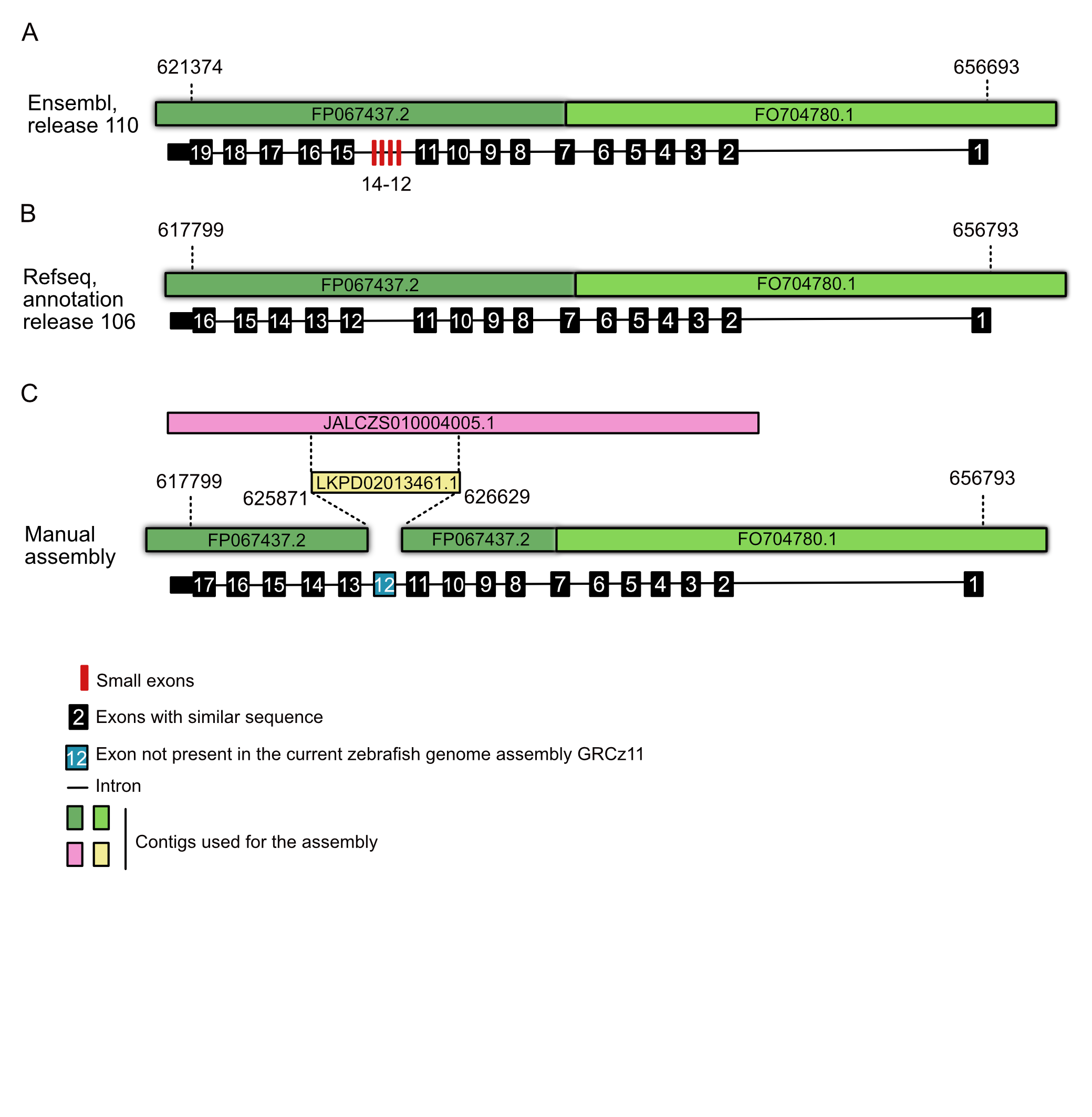

Supplement: Figure 8-1 — Outline of the gene assembly of the appa gene on Chromosome 1. The appa gene assembly in Ensembl (A), Refseq (B) and our manually assembled (C) adding contig LKPD02013461.1 to a region with di-nucleotide repeats in contig FP067437.2. The inclusion of this sequence is supported by contig JALCZS010004005.1 which cover flanking regions. Numbers and dotted lines indicated positions on chromosome 1. Download Figure 8-1, TIF file. [file eneuro-11-ENEURO.0034-24.2024-s005.tif]
